# Bacterial ectosymbionts in cuticular organs chemically protect a beetle during molting stages

**DOI:** 10.1038/s41396-022-01311-x

**Published:** 2022-09-02

**Authors:** Rebekka S. Janke, Filip Kaftan, Sarah P. Niehs, Kirstin Scherlach, Andre Rodrigues, Aleš Svatoš, Christian Hertweck, Martin Kaltenpoth, Laura V. Flórez

**Affiliations:** 1 Department of Evolutionary Ecology, Institute of Organismic and Molecular Evolution, Johannes Gutenberg University, 55128 Mainz, Germany; 2 Department of Insect Symbiosis, Max Planck Institute for Chemical Ecology, 07745 Jena, Germany; 3 Research Group Mass Spectrometry, Max Planck Institute for Chemical Ecology, 07745 Jena, Germany; 4 Department of Biomolecular Chemistry, Leibniz Institute for Natural Products Research and Infection Biology, HKI, 07745 Jena, Germany; 5 Department of Biochemistry and Microbiology, UNESP-São Paulo State University, Rio Claro, 13506-900 SP Brazil; 6 Institute of Microbiology, Faculty of Biological Sciences, Friedrich Schiller University Jena, 07745 Jena, Germany; 7 Department of Plant and Environmental Sciences, Section for Organismal Biology, University of Copenhagen, 1871 Copenhagen, Denmark

**Keywords:** Antibiotics, Zoology, Microbial ecology

## Abstract

In invertebrates, the cuticle is the first and major protective barrier against predators and pathogen infections. While immune responses and behavioral defenses are also known to be important for insect protection, the potential of cuticle-associated microbial symbionts to aid in preventing pathogen entry during molting and throughout larval development remains unexplored. Here, we show that bacterial symbionts of the beetle *Lagria villosa* inhabit unusual dorsal invaginations of the insect cuticle, which remain open to the outer surface and persist throughout larval development. This specialized location enables the release of several symbiont cells and the associated protective compounds during molting. This facilitates ectosymbiont maintenance and extended defense during larval development against antagonistic fungi. One *Burkholderia* strain, which produces the antifungal compound lagriamide, dominates the community across all life stages, and removal of the community significantly impairs the survival probability of young larvae when exposed to different pathogenic fungi. We localize both the dominant bacterial strain and lagriamide on the surface of eggs, larvae, pupae, and on the inner surface of the molted cuticle (exuvia), supporting extended protection. These results highlight adaptations for effective defense of immature insects by cuticle-associated ectosymbionts, a potentially key advantage for a ground-dwelling insect when confronting pathogenic microbes.

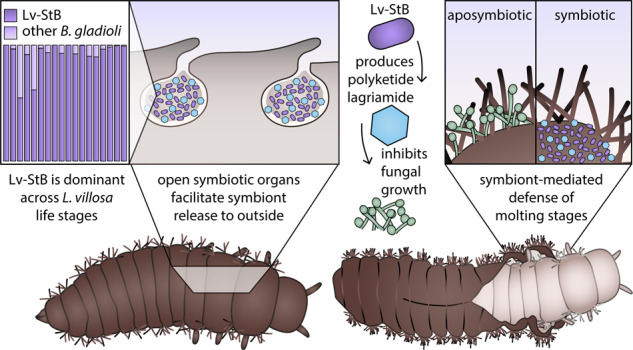

## Introduction

Fungal pathogens are important natural enemies of insects and exert strong selective pressures on populations in both natural and agricultural settings [1, 2]. Adhesion and germination of entomopathogens on the insect surface is followed by direct penetration of the cuticle and secretion of toxins, attacking a major protective barrier of insects [3]. In response, insects have evolved diverse defense mechanisms including elaborate immune responses, as well as behavioral, mechanical, and chemical defenses [4, 5]. While animals can often produce defensive compounds themselves, there is growing evidence that associated microorganisms can support protection against fungi and other enemies [6, 7]. Symbiotic microbes inhibit fungal infections in aphids [8, 9], eggs of *Lagria* beetles [10, 11], immature life stages of solitary wasps [12–14], fruit moths [15], and leaf-rolling weevils [16], as well as the nutritional resources of ants [17–19], termites [20] and ambrosia-beetles [21]. Although numerous compounds produced by defensive symbionts have been identified [6, 22–24], there are only a few systems in which these have been spatially tracked [25–28], since their quantification and detection in situ remains challenging.

Symbiont-mediated defense might be especially important for animals with temporarily limited protection mechanisms, like insects with complete metamorphosis, in which the distinct life stages —adult, egg, larva, pupa— face different challenges and demand specific defense strategies. Eggs are particularly susceptible to predation, parasitism, and pathogen infection since behavioral defenses are limited by the lack of mobility [29]. In *Lagria villosa* beetles, fungal infections are prevented by *Burkholderia* symbionts on the eggs, which produce different antimicrobial compounds [10, 11]. A similar strategy has been observed in squids [30], hoopoe birds [31], and lizards [32], indicating that egg protection through beneficial microorganisms has evolved in different environments for invertebrate and vertebrate hosts [33].

Insect larvae and pupae also face high infection risks, as they often inhabit pathogen-rich environments like soil or the phyllosphere. The leaf-rolling weevil *Euops chinensis* increases the survival of its larval offspring in leaf cradles by depositing the symbiotic fungus *Penicillium herquei* with the eggs [16]. Also in solitary beewolf wasps, antibiotic-producing *Streptomyces* bacteria are incorporated into the cocoon silk protecting the pre-pupa from mold fungi [12, 13]. Therefore, symbiont-mediated defense might be a common defense strategy for immature stages. The localization of microbial symbionts on the host surface or in connection to the external environment could be especially convenient to prevent infections. Cuticle-associated host defenses, including melanization [34, 35] and immune factors in the molting fluid [36, 37] might be thus complemented by ectosymbionts. However, immature insects recurrently shed their cuticle during development, which has both advantages and potential risks. Molting can clear out detrimental but also beneficial microbes from the surface, and it might lead to temporal vulnerability to antagonists until a fully sclerotized and melanized cuticle is reestablished [38, 39]. While these tradeoffs are likely common across many invertebrates that shed their cuticle, research on defense strategies during molting phases is lacking.

**Fig. 1:**
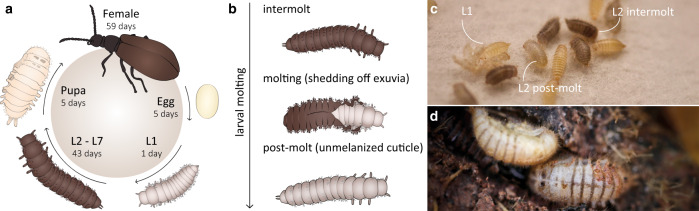
Life cycle of *L. villosa* beetles. **a** Schematic overview of the beetle’s life stages including average duration of every stage in days. The seven larval instars are abbreviated as L1–L7. **b** Illustration of the larval molting phase. **c** Photograph of young larvae reared in the laboratory. L1 larvae remain unmelanized, while the cuticle of L2 larvae melanizes after the post-molt phase. **d** Photograph of pupae in the field, found under decaying leaves in the soil of a soybean plantation.

In *L. villosa* beetles (Coleoptera: Tenebrionidae), ectosymbionts with high bioactive potential are associated with the insect throughout immature life stages [10, 11, 40] (Fig. 1a). This polyphagous beetle originates from sub-Saharan Africa and was introduced to South America, where it is found on several crop plants. Adults feed on leaves and flowers, while the larvae are mostly detritivorous [41]. Hence, *L. villosa* beetles occur in soil and around decaying plant material, where they deposit eggs, and the larvae feed, molt (Fig. 1b, c), pupate (Fig. 1d), and emerge as adults, demanding effective defense mechanisms against a variety of potential pathogens throughout their lifetime. Concordantly, the beetles harbor a community of bacterial symbionts including several strains of *Burkholderia gladioli* (Gamma-Proteobacteria, previously Beta-Proteobacteria), which are vertically transmitted from female accessory glands onto the egg surface during oviposition [10, 40]. Multiple strains can infect the beetles, and at least two, ﻿*B. gladioli* Lv-StA and ﻿*B. gladioli* Lv-StB (henceforth “Lv-StA” and “Lv-StB”, respectively), are capable of producing antimicrobial compounds that inhibit fungal growth on the beetle eggs [10, 11]. Under field conditions, Lv-StB is consistently present in high abundance within female accessory glands and on the egg surface, where it is important for defense presumably by producing the antifungal polyketide lagriamide [11]. The symbionts are absent in male adults but are present in both female and male larvae in the related species *L. hirta* [40], where they are housed as ectosymbionts in peculiar dorsal cuticular invaginations formed during embryonic development and so far described only in this insect genus [42].

Given the localization of the ectosymbionts in larvae in cuticle-lined organs of both sexes, we reasoned that, in addition to egg defense and transmission to the next generation, symbiont presence during larval development may be associated with the protection of molting stages. To test this, we investigated the functional role of antibiotic-producing *B. gladioli* symbionts in larvae and pupae of *L. villosa* beetles by (i) evaluating symbiont function in larval and pupal stages via bioassays against antagonistic fungi, (ii) characterizing and localizing the symbiont community in all life stages through 16 S rRNA gene amplicon sequencing, qPCR and FISH, and (iii) quantifying and localizing the bioactive secondary metabolite lagriamide in situ and monitoring its biosynthesis during development using ﻿high-performance liquid chromatography mass spectrometry (HPLC-MS) and ﻿mass-spectrometry imaging with a high-resolution atmospheric pressure scanning microprobe (AP-SMALDI-HR MSI). Thereby, we show prolonged antibiotic-mediated defense provided by a *Burkholderia* bacterial symbiont during the immature life stages of the beetles and highlight the symbiotic organs as unique host morphological adaptations that support the protective association.

## Results

### Symbiont-mediated defense against entomopathogens in early larval and pupal stages

**Fig. 2:**
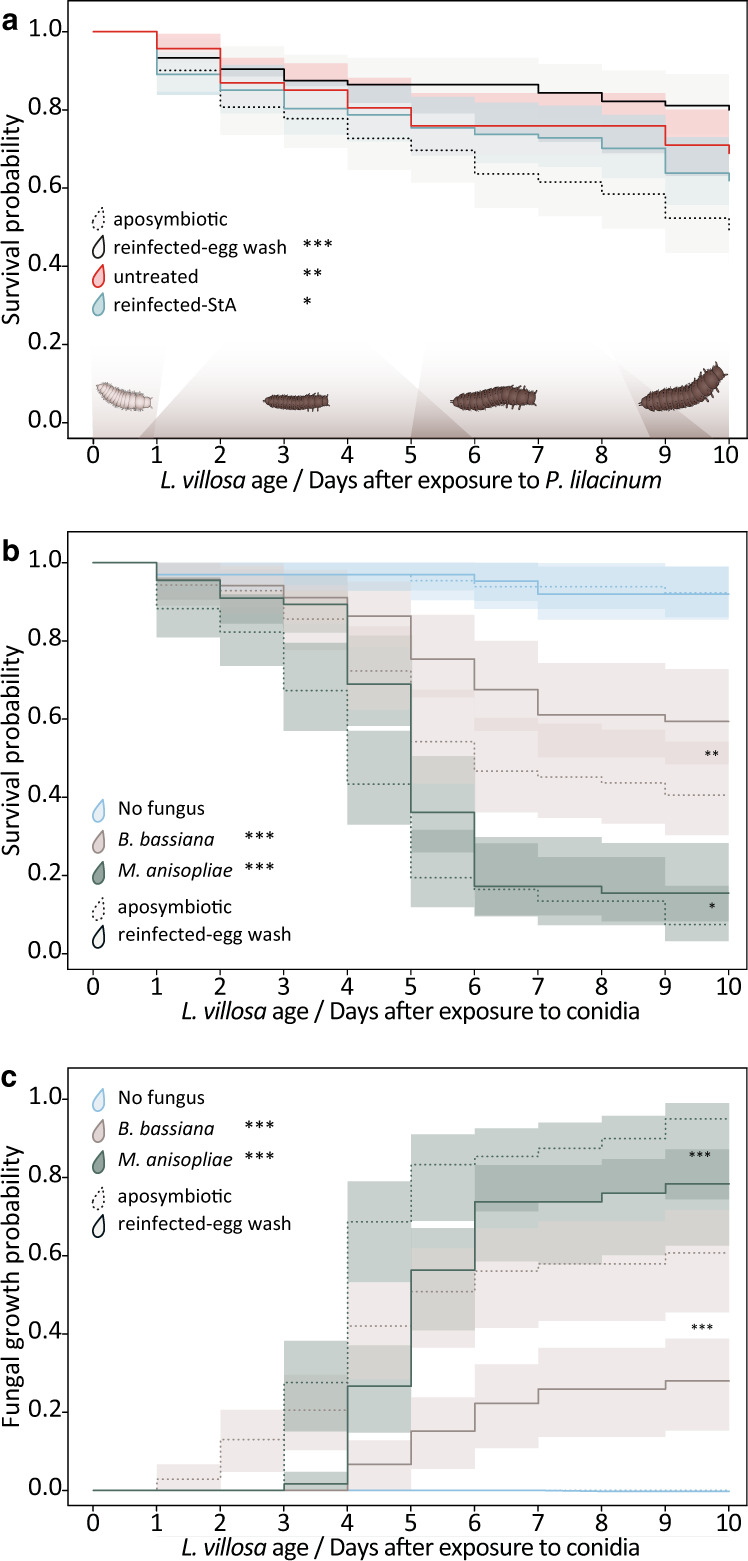
Bacterial symbionts reduce growth of pathogenic fungi and enhance survival of *L. villosa* larvae. 1st instar larvae with (solid lines) or without symbionts (dotted lines) were exposed to different fungal pathogens in small groups and were single-blind monitored for 10 days. **a** Survival was assessed for an environment with 7.5 × 10^3 ^*P. lilacinum* conidia in 460 larvae from 8 clutches. **b** Survival and (**c**) visible fungal infection was assessed for an environment with either 10^6^ conidia of *B. bassiana* (beige lines), *M. anisopliae* (dark green lines) or a no-fungus-environment (light blue lines) (in total 404 larvae from 4 clutches). Statistically significant differences in relation to aposymbiotic controls or no fungus controls (treatment legends in (**b**) and (**c**)) based on Cox mixed effects model: *p < 0.05, **p < 0.01 and ***p < 0.001. Estimated survival curves (Kaplan–Meier) and the corresponding 95% confidence intervals are shown.

To assess the potential protective function of the symbionts after egg hatching, we carried out bioassays exposing larvae to different pathogenic fungi and monitored survival and fungal growth (Table S1). We evaluated the first days of larval development covering multiple molting events since freshly molted larvae are likely more susceptible to infections [43]. First, we exposed young larvae to a fungal pathogen previously isolated from *L. villosa* beetles, *Purpureocillium lilacinum* [44]. In these conditions, aposymbiotic larvae were less likely to survive compared to symbiotic individuals including those that were untreated, reinfected with the natural symbiont community recovered from eggs, or with the culturable symbiont strain Lv-StA (Fig. 2a, Table S2, Cox mixed effects model, *p* values: untreated: 0.0017, reinfected-egg wash: <0.001, reinfected-StA 0.023), while there was no significant difference between untreated and reinfected larvae (Table S2, *p* value: 0.2). In a second assay, we focused on the natural symbiont community dominated by Lv-StB and tested its protective effect against two generalist entomopathogens—*Beauveria bassiana* and *Metarhizium anisopliae*– applying fungal titers comparable to amounts of fungal conidia in other studies and agricultural applications of entomopathogens [45–47] or natural soil [48, 49]. Because there was no difference between untreated larvae and larvae reinfected with the natural community in the previous assay, we compared symbiont-free larvae to reinfected larvae in the following assays, controlling for the surface-sterilization procedure [10]. To rule out the possibility that symbiotic larvae survived better because of a nutritional benefit by the symbionts, we added a control group that was not exposed to fungi. Again, when exposed to *B. bassiana* or *M. anisopliae*, symbiotic larvae were more likely to survive compared to aposymbiotic larvae (Fig. 2b, Table S3, Cox mixed effects model, *p* values: *B. bassiana*: 0.0028, *M. anisopliae*: 0.026). While the tested *M. anisopliae* strain was overall more virulent than that of *B. bassiana* and symbiont-conferred protection was weaker against *M. anisopliae* (Fig. 2b), the probability of fungal growth was significantly lower in symbiotic individuals for both fungi (Fig. 2c, Table S4, Cox mixed effects model, *p* values: *B. bassiana*: <0.001, *M. anisopliae*: <0.001), in line with a broad-spectrum defense. There were no differences in survival between symbiotic and aposymbiotic larvae in the absence of pathogenic fungi.

To test if symbionts can also protect pupae, we exposed aposymbiotic and symbiotic individuals directly after pupation to fungi and used two set-ups (Table S1). One set-up used a combination of different fungi (fungus-mix: *P. lilacinum, B. bassiana, M. anisopliae*) which was applied to the environment and the other set-up used a topical application of *M. anisopliae* conidia onto the dorsal thorax of pupae. When we exposed pupae to the fungus-mix or only to *M. anisopliae*, there was no difference in emergence rate (Fig. S1a) or survival of adults after emergence between the treatments (Fig. S1b, c). However, we observed melanization spots on the pupal surface, likely as a reaction to fungi, which was significantly higher in aposymbiotic individuals infected with *M. anisopliae* (Fig. S1d, Cox proportional hazards regression model, *p* value = 0.037). Additionally, we observed a higher probability of visible fungal growth on pupae infected with *M. anisopliae* (Fig. S1e, Cox proportional hazards regression model, *p* value = 0.012). Although adult emergence rate and survival after emergence were not different between both groups infected with fungi (aposymbiotic and untreated), all emerged adults died within eight days (Fig. S1c) which is not expected given the usual longevity in absence of fungal infections (Fig. 1). This might be due to the high exposure to the pathogen, and/or the gradual decline in Lv-StB titers under laboratory conditions, which has been previously documented [40, 50]. Foreseeing this problem, we maintained the symbiotic individuals on soil and live plants, which decreases the risk of symbiont loss [50]. However, only three out of five female pupae tested before the experiment still carried *Burkholderia* spp., and none of the adults that emerged (Fig. S1f). In summary, the natural symbiont community present in the dorsal organs and on the insect’s surface inhibited fungal infections in young larvae and pupae and increased the chances of larval survival in the presence of fungal pathogens.

### Symbiont strain Lv-StB is maintained in special dorsal cuticle invaginations and becomes dominant during *L. villosa* development

**Fig. 3:**
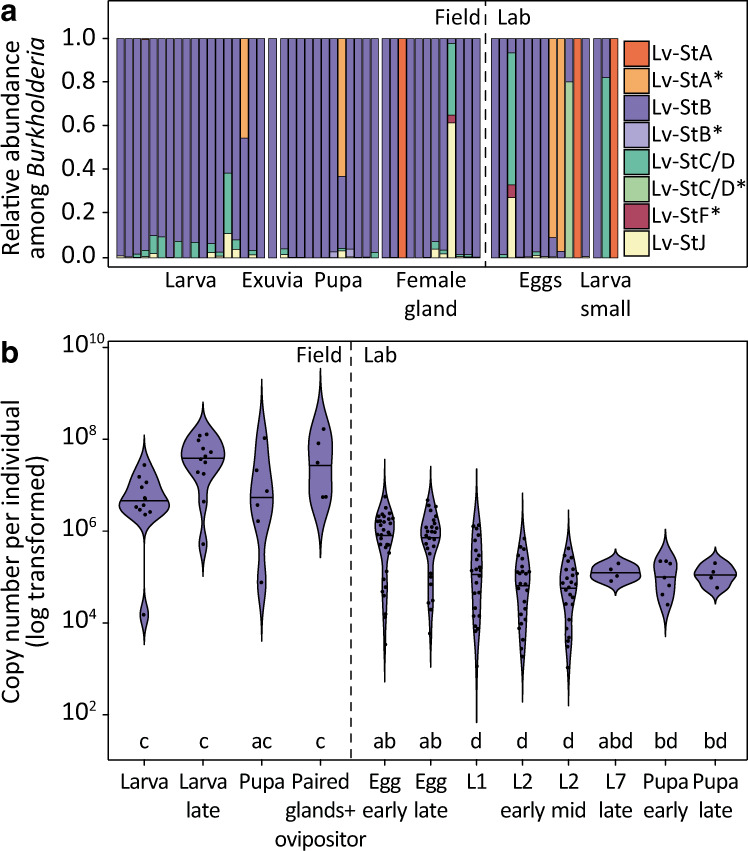
*Burkholderia* Lv-StB abundance throughout host development. **a** Relative abundance of *Burkholderia* ASVs across different life stages based on Illumina sequencing of the 16 S rRNA gene V4 region compared to whole genome or MAG references of previously described strains. Each bar corresponds to a single individual, dissected organ of an adult female (“female gland”) or egg clutch. An asterisk (*) in the legend denotes pairwise identity above 98% but below 100%. **b** Lv-StB titers across host development in field and laboratory-raised individuals (Table S5) measured by qPCR with primers targeting the single-copy trans-AT PKS/NRPS lgaG gene involved in the biosynthesis of lagriamide by Lv-StB. Different letters indicate significant differences between life stages (Kruskal-Wallis χ2 = 97.712, df = 11, *p* value = 5.1 × 10^−16^, posthoc Dunn’s Test, α ≤ 0.05).

To evaluate if protection in larval and pupal stages is mediated by the same symbionts as previously described in eggs, we investigated the bacteria associated to field-collected *L. villosa* individuals and tested whether Lv-StB dominates the community beyond the egg stage (Fig. 1a). As in female glands and on the egg surface [10], Burkholderiaceae were consistently present in larvae and pupae (Fig. S2a). While Enterobacteriaceae and Rhizobiaceae were also predominant in larvae, the profiles correspond to the full body, and these are therefore most likely associated with the gut. As observed directly by in situ localizations using microscopy, *Burkholderia* is present and becomes dominant in the dorsal structures of older larvae (Fig. S3). Lv-StB is particularly predominant in the community, being present in 95% of the samples and showing the highest relative abundance among *Burkholderia* ASVs (Amplicon Sequence Variants) in 82% of the 58 samples (Fig. 3a). The three individuals in which Lv-StB was not detected showed high relative titers of the ASV assigned to the culturable strain *B. gladioli* Lv-StA, or a closely related ASV (Fig. 3a, Fig. S2b). In early larvae, Lv-StB was present in very low abundance, judging from the absolute read counts in these samples (Fig. S2b) and FISH (Fig. S3a–c). While this might result from a bottleneck during vertical transmission or natural variation among individuals, we cannot rule out that the low titers are associated with laboratory conditions, since *Burkholderia* symbionts are often lost during controlled rearing [40, 50]. To identify potential differences in absolute symbiont abundance at a fine temporal scale during development, we quantified Lv-StB symbiont titer using qPCR across larval and pupal stages. Additionally, we considered both field-collected and laboratory-raised individuals for the life stages in which this was feasible (Fig. 3b). The mean abundances of Lv-StB copy numbers were the highest in females and tended to decrease in laboratory-raised individuals as host development advanced. As expected [40], Lv-StB titers of later timepoints were higher in field-collected individuals than in laboratory-raised individuals. Taken together, the results suggest that Lv-StB is the dominant *Burkholderia* strain across larval development and is consistently present throughout the life cycle (Fig. 3a). Additional involvement of other bacteria in protection is also possible, given the consistent presence of specific groups, albeit in lower relative abundance in symbiotic organs.

**Fig. 4:**
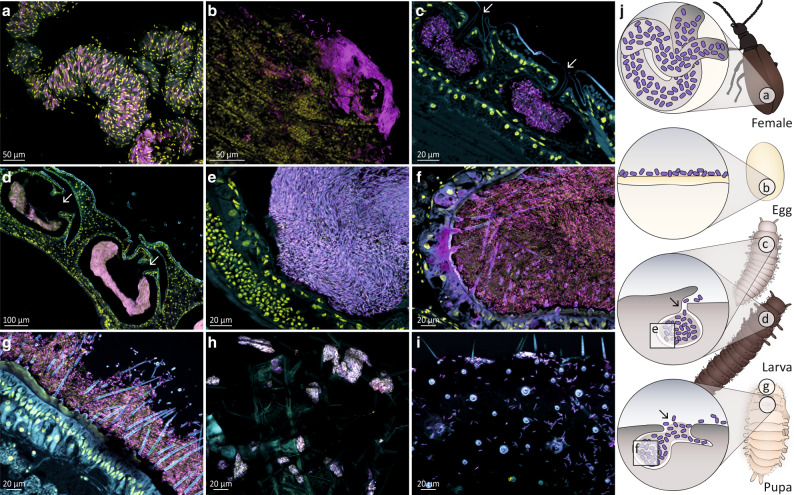
*Burkholderia* Lv-StB symbiont localization and prevalence throughout the different life stages of *L. villosa* using fluorescence in situ hybridization. Symbiont cells are generally depicted in magenta, and host cell nuclei in yellow. **a** Whole mount of a female abdomen showing a dense population of Lv-StB in tubes of the accessory glands. **b** Whole-mount of an egg revealing Lv-StB dominance. **c** Sagittal section of a 1st instar larva revealing a dense culture of Lv-StB in the dorsal symbiotic structures as well as an opening to the external environment (white arrows). **d** Sagittal sections through the three pouches of a later larval stage showing the same morphology and (**e**) the dominance of Lv-StB inside the pouch. **f** 1st dorsal symbiont compartment and (**g**) outer surface of a pupa showing dominance of Lv-StB. In (**a**–**g**) *B. gladioli* specific staining is shown in cyan (Burk16S_Cy3), Lv-StB-specific staining in magenta (Burk16S_StB_2_Cy5), and host cell nuclei in yellow (DAPI). **h** First larval exuvia covered with *B. gladioli*. **i** Inner side of a larva-pupa exuvia with visible symbionts cells. FISH on exuviae (**h**, **i**) show general eubacterial staining in cyan (EUB338_Cy3) and *B. gladioli* specific staining in magenta (Burk16S_Cy5). **j** Schematic guide illustrating symbiont localization throughout *L. villosa* development. 1st instar larva individuals are from the 1st lab generation, and all other individuals are field collected. In every image autofluorescence of the host tissue is shown in cyan and overlap of all three channels is shown in purple-white. Arrows indicate the opening of the symbiotic structures in larvae and pupae. Scale bars correspond to 20 µm, except for panels a (50 µm) and (**d**) (100 µm).

To localize Lv-StB in each life stage and further assess its dominance within the symbiotic organs, we carried out fluorescence in situ hybridization (FISH) with specific probes targeting this strain (Figs. S4, S5) on histological sections throughout host development (Fig. 4). After symbionts are transmitted from female accessory glands (Fig. 4a) to the egg surface (Fig. 4b), embryos develop three equally sized organs located dorsally, which likely originate as invaginations of the cuticle. From the first larval instar on (L1, Fig. 4c), the pouches are filled with symbionts. These peculiar organs remain open to the outside through a small cuticle-lined canal and are maintained throughout larval development, increasing in size as the insect grows [42] (Fig. 4d, e). In female pupae, symbionts can be found in the first dorsal pouch (Fig. 4f) and on the surface of the head region between bristles (Fig. 4g). To evaluate if ectosymbionts are released from the pouches, we carried out FISH on whole exuviae of the first (L1) and last larval instar (larva-to-pupa molt). We detected *Burkholderia* symbionts on the external surface of L1 exuviae (Fig. 4h), which might be explained by contact with the egg chorion during hatching. We also found high amounts of *Burkholderia* on the internal surface of larva-to-pupa exuviae (Fig. 4i) and the outside surface of pupae, as noted above (Fig. 4g). These findings indicate that the symbionts not only colonize the dorsal organs but are also present on the surface of early larvae and pupae. The probe used to target Lv-StB cells showed low-intensity unspecific labeling on a pure Lv-StA culture (Fig. S4 a, b), but a direct comparison on an egg wash (Fig. S4c, d) and tissue sections (Fig. S5) confirmed much more prominent labeling of Lv-StB cells, supporting the results observed by sequencing. In summary, Lv-StB is the most prevalent bacterial strain in symbiotic pouches during the life cycle of *L. villosa*, and symbionts are also released to the surface of larvae and pupae, as indicated by their presence on exuviae and pupal bristles.

### Presence of lagriamide throughout the host life cycle and bioactive potential

**Fig. 5:**
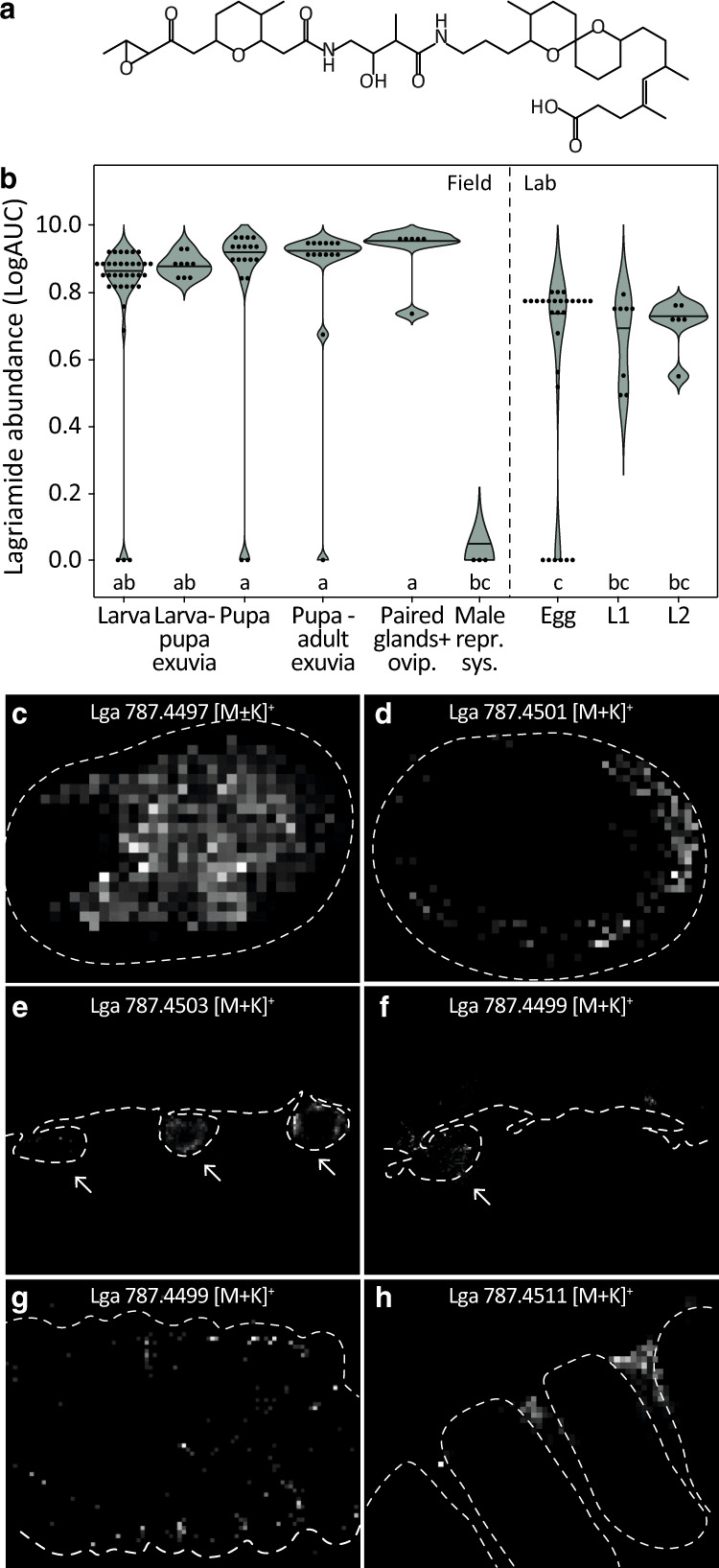
Lagriamide is present across *Lagria villosa* life stages and co-localizes with Lv-StB in regions exposed to the external environment. **a** Chemical structure of lagriamide [11]. **b** Area under curve (AUC) of the extracted ion chromatogram (EIC) of lagriamide (*m/z*  =  747.4769–747.4843 [M-H]^−^) representing abundance across host development (Table S6), as quantified from crude methanol extracts. Eggs, as well as first (L1) and second (L2) instar larvae correspond to offspring from field-collected females. All others correspond to field specimens. Different letters indicate significant differences between life stages (Kruskal-Wallis χ2 = 66.988, df = 8, *p* value = 1.95e–11, posthoc Dunn’s Test, α ≤ 0.05). **c**–**h** 2-D ion maps obtained by AP-SMALDI-MSI representing the potassium adducts of lagriamide [M + K]^+^ across *L. villosa* life stages. **c** Surface analysis of an intact egg and (**d**) an egg cryosection showing lagriamide presence on the surface. **e** In larval sections, lagriamide is present inside the symbiotic organs (arrows). **f** In pupal sections, lagriamide is mainly detected in the first symbiotic organ (arrow). **g** On the inner surface of a larva-to-larva exuvia, lagriamide is either scattered over the thoracal segments or (**h**) distinctly located between the thoracic segments and first abdominal segment, which corresponds to the location of the symbiotic organs. Dotted lines were manually added based on corresponding light microscopy pictures (Fig. S6) to indicate specimen profiles.

As shown in Fig. 4, Lv-StB is highly abundant in larvae and pupae, the symbiotic pouches are connected to the outer surface and the symbionts are present on the outer and inner surface of exuviae. To gain further insight into the chemical basis and dynamics of defense observed in early larval and pupal stages, we investigated whether the Lv-StB bioactive secondary metabolite lagriamide (Fig. 5a) is also produced during these time points and where it is located in the host. We first analyzed crude extracts of field-collected individuals and offspring of field-collected females by HPLC-HRESI-MS and evaluated the presence of lagriamide by comparing retention time, UV absorbance, and high-resolution mass spectra to an authentic reference. We detected lagriamide in every life stage except in adult males (which lack symbionts), showing that lagriamide is consistently produced across symbiont-bearing life stages of *L. villosa* and is also present on shed exuviae (Fig. 5b). Using AP-SMALDI-HR MSI, we localized lagriamide on the egg surface, in larval and pupal symbiotic organs as well as distributed across the surface of exuviae from larval molts (Fig. 5c–h, Fig. S6), whereas we could not detect it on individuals without the natural symbiont community (Fig. S7). Thus, lagriamide co-localizes with Lv-StB throughout host development, i.e., in female accessory glands, on the egg surface, in symbiotic organs of larvae and pupae, and on exuviae. Although we did not detect lagriamide on the surface of larval sections via AP-SMALDI-profiling, possibly because the abundance resulting after histological sectioning is below the detection limit, HPLC-MS, and AP-SMALDI-profiling confirmed its presence on intact molted exuviae, and it spatially coincided with Lv-StB presence. Additionally, in vitro activity profiling of pure lagriamide revealed that it hinders the growth of *B. bassiana* and *M. anisopliae* on plate, showing partial inhibition (Fig. S8a, b). This observation is in line with the previously reported antifungal activity against *P. lilacinum* and *Aspergillus niger* [11], and the larval antifungal assays (Fig. 2). However, we did not observe antibacterial effects against the entomopathogen *Bacillus thuringensis* under these conditions (Fig. S8c). Taken together, these findings indicate that the dominant symbiont strain Lv-StB produces the antifungal polyketide lagriamide throughout the developmental stages of *L. villosa* from eggs to pupae.

## Discussion

*L. villosa* beetles are associated with a bacterial community dominated by *Burkholderia* [10] harbored in specialized symbiotic structures only absent in male adults. The symbionts are vertically transmitted, although sporadic acquisition from the environment likely occurs [9, 42]. Multiple *Burkholderia* strains can infect the beetles, occasionally simultaneously [40], and at least two —Lv-StB and Lv-StA— are known to produce antimicrobial compounds inhibiting fungal growth on the insect’s eggs and thereby enhance survival [10, 11]. Here, we show consistent presence of the antimicrobial lagriamide and its producer Lv-StB throughout the host life cycle. In situ localization of both Lv-StB and lagriamide confirms their presence within open symbiotic organs and their distribution across the surface of shed exuviae, demonstrating release of symbionts and the protective compound during larval molting. Reduced fungal growth and enhanced survival of young larvae in presence of Lv-StB, when confronted with different fungi and in vitro antifungal activity of pure lagriamide against fungi, demonstrate the protective role. These findings reveal a case of host adaptation to house ectosymbionts in specialized cuticular organs, which facilitate external release of bacteria and the associated compounds that aid in host protection. At the same time, these organs offer a suitable environment for growth and maintenance of antibiotic-producing ectosymbionts despite recurrent molting.

Like other arthropods living in the soil, *L. villosa* larvae and pupae face the challenge of fungal pathogens in the environment. This risk is particularly high in phases with reduced structural protection due to an incompletely sclerotized cuticle after molting [38, 39]. The cuticle is a key protective barrier against pathogenic fungi in almost all arthropods because of its rigidity [3], but also through the presence of antifungal proteins (e.g. glucanases, or proteinase- and chitinase-inhibitors), or small molecules with inhibitory activity, like cuticular fatty acids or benzoquinones [43, 51, 52]. For example, *Galleria mellonella* morphs with higher amounts of melanin, increased ﻿dihydroxyphenylalanine (DOPA) decarboxylase activity but lower amounts of hydrocarbons show reduced fungal attachment and germination, resulting in increased host survival in comparison to weakly melanized larvae [53]. Yet, relying on the cuticle as a primary armor can involve important tradeoffs. While the phenomenon of molting practiced by ecdysozoans, including arthropods and nematodes, enables growth or reaching the next life stage, it also entails the cost of a temporarily softer and thinner integument. Right after molting, individuals might therefore be more susceptible to natural enemies, as described for some crustaceans [54, 55]. However, molting itself can shed off epibionts [56] or pathogens that were not able to penetrate the cuticle. In the crustacean *Daphnia magna*, for example, the bacterial pathogen *Pasteuria ramosa* has lower chances of a successful infection when the host molts soon after exposure to the pathogen [57]. Also, in the diamondback moth *Plutella xylostella*, larvae inoculated with fungi early during an instar are more likely to die than if inoculated later during the same instar [58]. It is also possible that fungi can breach the cuticle during vulnerable periods and proceed with infection within the insect host even after several molting events, as occurs in larvae of Colorado potato beetles [59]. Taken together, such findings in different arthropods indicate that while the cuticle itself is the first major barrier against pathogens and molting can remove both beneficial and harmful microbes from the surface, the early period after ecdysis is particularly vulnerable due to a softer cuticle and/or the longer period available for pathogens to colonize and establish before the next molt.

Despite a long history of research on defense mechanisms of arthropods against fungal pathogens [3], the vulnerability of molting stages and adaptations for protection in these phases have rarely been studied, particularly in insects. However, studies in *Bombyx mori* and *Tribolium castaneum* show that the molting fluid that is secreted and accumulates during molting between the old and new cuticle [60] not only enables successful ecdysis but could also protect vulnerable postmolt insects against microbial pathogens [37]. In *L. villosa*, critical phases where structural protection is limited are the first larval instar, which remains unmelanized for around 24 h, and the phases after each of the seven larval molts and during pupation, where individuals also lack mobility. By exposing symbiotic and aposymbiotic individuals to pathogens over the first 10 days after hatching and during pupation, respectively, we covered multiple molting events as well as metamorphosis and showed that cuticle-associated symbionts can persist despite recurrent molting and provide an effective means for protecting these life stages against fungal pathogens. Our setup does not directly demonstrate that fungal entry is occurring immediately after ecdysis in aposymbionts, yet the presence of both the symbionts and the antifungal lagriamide over extended areas of the inner surface of shed exuviae are in line with their role as a protective barrier to infections upon molting. In pupae, our results suggest a moderate inhibition of fungal growth by the symbionts, although we did not find a strong advantage in pupal emergence or survival shortly after emergence. This might be associated to the potential loss of symbionts after some time under laboratory conditions in untreated individuals, as previously reported for this insect [40], or an alternative line of defense in the pupal stage. As aposymbiotic beetles that are not challenged with fungal infections can fully develop and lay eggs in the laboratory [10], it is challenging to manipulate and individually track the symbiotic status of *Lagria* beetles up to the adult stage. However, the inhibition of fungal growth on pupae, the high abundance of Lv-StB symbionts on the pupal surface, and lagriamide detection inside the enlarged dorsal organ are in line with symbiont-mediated protection during pupation. Altogether, our results reveal symbiont-provided protection against fungal pathogens as an adaptive defense strategy of immature insect life stages, which likely supplements existing immune responses and structural defenses.

The evolution of the protective symbiosis in *Lagria* beetles likely relied on the dorsal cuticular invaginations as a morphological adaptation facilitating accommodation and release of ectosymbionts. The external location of symbionts makes defensive metabolites readily available for deterring antagonists that attack insects from the exterior [61], while the crypt-like structures grant shelter to symbionts and likely provide nourishment from surrounding glandular cells [42]. Similarly, attine ants carry defensive *Pseudonocardia* bacteria in cuticular crypts on their surface, which protect both ants and their fungal gardens against pathogens [62–64]. Another structurally and functionally similar organ is found in solitary beewolf wasps, which harbor *Streptomyces* bacteria in cuticle-lined reservoirs of their antennae that protect the developing larva by producing an antibiotic cocktail [12, 13, 65, 66]. Also, ectosymbionts of marine ciliates defend their host against predators, and bacterial symbionts on developing embryos of certain shrimps and lobsters can inhibit infections by pathogenic fungi [67, 68]. Cuticular organs that foster symbiont colonization are also described in several other insects, and fungus-bearing mycangia of wood-boring and bark beetles, as well as leaf-rolling weevils, resemble pouches of *L. villosa* larvae [16]. Interestingly, although the pouches of *L. villosa* larvae are invaginations of the cuticle and are connected to glandular cells, they are not shed off with the exuvia as usually seen in ﻿exocrine glands of insects [69]. This prevents a complete loss of symbionts during molting, which is also reflected by similar symbiont titers from larvae after molting and intermolt stages (Fig. 3b, comparison between early L2 and mid L2). Similarly, other molting arthropods like nymphs of the phasmid *Oreophoetes peruana* do not discard the cuticular lining of their defensive glands during molting, conserving the costly secretion and keeping it available as a chemical defense directly after molting [70]. The external location of the symbionts might be additionally favorable for the host to avoid contact with potentially harmful bioactive secondary metabolites produced by the bacteria [6].

Although it was previously demonstrated that multiple *Burkholderia* strains (including Lv-StA) can coinfect single *L. villosa* beetles [10] and FISH revealed occasional presence of other bacteria and strains in the organs of younger larvae (Fig. S3a–c), Lv-StB is generally predominant and shows signs of a tight association with its host. Possibly, host provisions support its proliferation and enable it to remain in the organs despite its reduced genome [71]. The dominance of this strain also underlines the putative importance of the bioactive polyketide lagriamide for host protection or competitive exclusion of coinfecting microorganisms. However, other *Burkholderia* strains and community members might also contribute to host protection, as they were either shown to be capable of producing bioactive metabolites [10, 72, 73] or to carry gene clusters for putative defensive molecules [71]. Such coinfections by closely related symbiont strains in a single individual as observed in *L. villosa* are rare in insects, or so far understudied. However, defensive symbioses are often described as dynamic and can be shaped by multiple coinfections throughout a population or by symbiont replacements [6], offering an opportunity to acquire multiple defensive partners. Like other insects harboring defensive symbionts with a broad bioactive potential [12, 13], *Lagria* beetles are not challenged by a single antagonist, but rather by a variety of pathogens in the soil. Having the possibility for symbiont uptake via a mixed-mode symbiont transmission and simultaneously a tight association to one specific defensive symbiont strain, the beetle might have evolved a flexible strategy to adapt to various antagonists. Thus, the protective effect of the dominant symbiont Lv-StB through the production of lagriamide may be complemented by other compounds of associated microorganisms.

In conclusion, we show that in *L. villosa*, the adaptations to house antifungal-producing *Burkholderia* bacteria in cuticular invaginations in larvae and pupae enable defense against entomopathogens throughout development including multiple molting phases. These structures foster a specialized association with a specific defensive symbiont strain while maintaining flexibility to acquire additional protective strains from the environment. In contrast to highly intimate nutritional symbioses in which morphological adaptations such as bacteriomes have evolved, defensive symbioses usually have intrinsically different constraints regarding where and how microorganisms are maintained for effective protection, especially if deploying bioactive compounds. Cuticle-associated ectosymbionts can therefore be an effective and dynamic defense strategy influencing antagonistic interactions between arthropods and pathogenic microbes.

## Materials and methods

### Insect collecting and rearing

*L. villosa* individuals were collected in plantations of various crops in the states of São Paulo and Paraná, Brazil, from 2017 to 2020 (Table S7). Individuals were reared in plastic containers in a climate chamber (16:8 L:D light regime with 0.5 h dusk, 26 °C, and 60% humidity). Adult beetles were fed with iceberg lettuce, soybean, and kidney bean leaves, and larvae were fed with dry soybean and kidney bean leaves. Centrifuge tubes with autoclaved tap water and cotton on top and centrifuge tube lids with moist cotton were provided for humidity control and as egg-laying substrate.

### Bacterial community profiling via 16 S rRNA gene amplicon sequencing

*L. villosa* specimens were collected directly from the field or recovered in the lab as offspring of field-collected females in case of eggs and small larvae and preserved in ethanol or methanol. Accessory glands of female specimens were dissected before DNA extraction. Eggs were extracted as full clutches or a fraction of a single clutch (min. 18 eggs per clutch), while small larvae were extracted individually and later pooled by clutch combining DNA from either three (10 days old) or two (15 days old) individuals. All other samples were extracted individually. The Epicenter MasterPure Complete DNA and RNA Purification Kit was used for DNA extraction of a total of 58 samples following the manufacturer’s instructions and including lysozyme treatment before protein digestion. The V4 region of the 16 S rRNA gene was sequenced by a commercial provider (StarSeq, Mainz, Germany) on a MiSeq platform (Illumina), using primers 515 F [74] and 806bR [75, 76], double indexing, and a paired end approach with a read length of 300 nt. Amplicon sequence variants (ASVs) were inferred using the R package DADA2 [77] with default parameters including dereplication, chimera removal, and trimming lengths of 250 and 140 nt for forward and reverse reads, respectively. This corresponded to a 253 bp fragment of the V4 region. Taxonomy was assigned using the pre-trained classifier SILVA 132 [78, 79] with subsequent removal of reads classified as chloroplast or mitochondria. For graphical representation, only ASVs were shown that reached 1% or more of the reads per sample, in at least one sample. ASVs within *Burkholderia*ceae classified as “*Burkholderia*-*Caballeronia*-*Paraburkholderia*” or not assigned at the genus level were evaluated manually to detect previously described *Burkholderia* symbiont strains [11, 71], for which whole-genome or Metagenome-assembled Genome (MAG) references are available. In the evaluated region, Lv-StB shows a pairwise dissimilarity of at least 9 nucleotides with respect to the other reference sequences in the database. Four of the obtained ASV sequences had a pairwise identity of 100% to a sequence in the local database and another four ASVs were between 98 and 99.6% in similarity. These ASVs were assigned accordingly. All others were below 93% in similarity to the reference sequences in the database.

### Quantification of Lv-StB throughout beetle life stages

Different developmental stages (Table S5) of *L. villosa* individuals from the field and the 1st lab generation were collected in replicates and frozen at –80 °C before nucleic acid extraction. Females were dissected beforehand to obtain only the symbiont-bearing structures. Samples were homogenized with liquid nitrogen and nucleic acids were extracted using the innuPREP DNA/RNA Mini Kit (Analytik Jena) following the manufacturer’s guidelines. qPCRs were carried out with the DNA targeting the trans-AT PKS/NRPS lgaG region of the lagriamide gene cluster with the specific primers LgaG_3_fwd (CGCCGTATCGAGCAGTTTC) and LgaG_3_rev (CAACTGGTCGAGCGTATCAA) under the following conditions: Initial activation at 95 °C for 15 min, denaturation at 95 °C for 15 s, annealing at 65 °C for 15 s and elongation at 72 °C for 15 s. qPCRs were carried out using the 5x HOT FIREPol EvaGreen HRM Mix EvaGreen (Solis BioDyne) on a RotorGene-Q cycler (Qiagen) in 10 µL reactions including 0.5 µL of each primer and 1 µL template DNA. Standard curves were generated by amplifying the fragment, followed by purification and determination of the DNA concentration using a Qubit fluorometer (Thermo Fisher). Afterwards, a standard containing 1 ng/µL was generated and 1:10 serial dilutions to 10^−8 ^ng/µL were prepared. All standards were included in the qPCR run for absolute quantification. qPCR copy numbers were corrected for the number of individuals extracted in each sample (Table S5). Influence of the different life stages on the symbiont titer was analyzed by a Kruskal-Wallis and a posthoc Dunn’s test using the PMCMRplus package (Version 1.9.0) in RStudio (Version 1.2.5042). Plots were created using the ggplot2 package (Version 3.3.0) and Adobe Illustrator (Adobe, Version 14.1, CC 2020).

### Symbiont localization by fluorescence in situ hybridization (FISH)

FISH was either performed on entire tissue (whole-mount), on semithin sections of *L. villosa* individuals, on an egg wash, or pure symbiont culture (Lv-StA). Either field-collected individuals (female, bigger larvae, pupae, larva-pupa-exuvia) or offspring of field-collected females (egg, L1 larva, L1 exuvia) were used. Before FISH, *L. villosa* individuals and exuviae were fixed in 4% formaldehyde for at least 3 days, while the egg wash and symbiont culture were fixed on glass slides with ethanol. Embedding, semithin sectioning, and FISH were performed as described previously [80] using a hybridization temperature of 50 °C. The Cy3- or Cy5-labeled Burk16S probe (5ʹTGCGGTTAGACTAGCCACTʹ3) was used to mark all *Burkholderia* strains, and the Cy5-labeled Burk16S_StB_2 probe (5ʹGGCAACCCTTTGTTTTGACCʹ3) was used for the symbiont strain Lv-StB, Cy5-labeled Burk16S_StA probe (5ʹGCACCCTCAGATCTCTCCAAGG3) was used for symbiont strain Lv-StA and Cy3-labeled EUB338 probe [81] (5ʹGCTGCCTCCCGTAGGAGTʹ3) was used for general eubacteria. DAPI (40,6-diamidino-2-phenylindole) was used to label the host cell nuclei and as counterstaining. Images were taken on an AxioImager.Z2 fluorescence microscope (Zeiss, Jena, Germany).

### In vivo bioassays with L. villosa larvae

Larval bioassays (Table S1) were carried out at 26 °C in small Petri dishes prepared as follows: Sterile vermiculite (1–2 mm) was distributed over the bottom of the dish and humidified with sterile water. Sterile filter paper inoculated either with 100 µL sterile water (no-fungus-treatment) or 100 µL of a fungal conidial suspension in sterile water (75 conidia/µL of *P. lilacinum* (LV1, Accession number: KY630747, KY630748, KY630749), or 10^4^ conidia/µL of *B. bassiana* (LESF477, Database Identifier: CRM 1216, CRM - UNESP) or *M. anisopliae* (LESF 206, Database Identifier: CRM 530, CRM - UNESP), respectively) was added. In each dish, maximum five 1^st^ instar larvae were placed according to their treatment and single-blind monitored for 10 days. UV-sterilized dried soybean and kidney bean leaves were provided as food sources every other day. To obtain aposymbiotic and differently reinfected 1^st^ instar *L. villosa* larvae, 1–2 day(s) old egg clutches of field-collected females were divided into different groups. One part of the clutch was used as a control with the natural microbial community (untreated). Remaining eggs were first washed with sterile PBS, slightly shaken for 5 min in 70% ethanol, washed 2 times with sterile water, immersed for 30 s in 12% NaClO, and washed three times with sterile water. Eggs were then inoculated with PBS to obtain symbiont-free individuals (aposymbiotic), with the previously obtained egg-wash that contains the natural microbial community (reinfected-egg wash, previously named “reinfected-natural” [10]), or with a PBS suspension of a cultured symbiotic strain of *L. villosa* beetles (10^6^ cells/µL) — *B. gladioli* Lv-StA (reinfected-LvStA). While this strain is only occasionally found in the beetles in natural conditions, we included it in this first assay to evaluate its protective potential, since it has been previously shown to chemically inhibit fungal growth on *L. villosa* eggs [10]. The *P. lilacinum* assay was carried out with larvae from eight clutches laid by different *L. villosa* field-collected females in two consecutive years. Survival of hatching larvae was observed for 10 days in a single-blind assay. *B. gladioli* and Lv-StB presence on the egg clutches was verified via qPCR using *B. gladioli* specific primers Burk16S_1_F ﻿(GTTGGCCGATGGCTGATT) and Burk16S_1_R ﻿(AAGTGCTTTACAACCCGAAGG) and Lv-StB specific primers LgaG_3_fwd and LgaG_3_rev on the five clutches from the 2020 population. The corresponding qPCR reactions were carried out under the following conditions: Initial activation at 95 °C for 15 min, denaturation at 95 °C for 15 s, annealing at 65 °C for 15 s and elongation at 72 °C for 15 s.

The assay including *B. bassiana*, *M. anisopliae*, and the no-fungus-control was carried out with four clutches laid by different *L. villosa* field-collected females comparing aposymbiotic and reinfected-egg wash individuals. Given the inconsistent occurrence of Lv-StA in natural conditions and giving priority to the naturally dominant strain Lv-StB, a Lv-StA reinfection treatment was not included in this assay to guarantee a robust sample size per group. After hatching, larvae were observed for 10 days monitoring survival, developmental time, and fungal infection in a single-blind assay. To assess fungal growth, larvae were monitored under a stereoscope for visible fungal hyphae and sporulating fungus on the larval surface. Fungal and bacterial strains were grown on King B Agar plates or King B liquid medium. Symbiont presence of Lv-StB on the original eggs and larvae was verified via qPCRs of DNA extracts from a subset of untreated eggs and surviving larvae from each treatment using Lv-StB specific primers LgaG_3_fwd and LgaG_3_rev.

To determine the effect of different treatments on survival and fungal growth probability on *L. villosa* larvae, a Cox mixed effects model with treatment and fungus as fixed effect and a random intercept per clutch and per year was fitted using the coxme package (Version ﻿2.2-16) in RStudio (Version 1.2.5042) (Table S2-S4). Plots were obtained with the rms package ﻿(Version 5.1-4), making use of the Kaplan–Meier-estimator, and Adobe Illustrator (Adobe, Version 14.1, CC 2020).

### In vivo bioassays with L. villosa pupae

The pupal bioassays (Table S1) were carried out at 26 °C with no light in 24 well-plates. Each well was prepared with soil that was inoculated with 100 µL of fungus suspension 2–4 days before the experiment. The fungus mix for the first assay contained 10^6^ conidia of each of the three fungi *B. bassiana*, *M. anisopliae*, and *P. lilacinum*. For the second assay, 10^6^ conidia of *M. anisopliae* were inoculated into the soil and additional 10^6^ conidia were inoculated onto the dorsal thorax of the pupae directly after pupation. Aposymbiotic and untreated pupae from the first lab generation were randomly placed in each well as soon as they emerged. Melanization and fungal growth were observed single-blind until emergence. Aposymbiotic individuals were obtained through the egg sterilization procedure described above and kept in a semi-sterile box until pupal emergence (around seven weeks). Symbiotic individuals were reared in a small terrarium on natural soil with growing soybean plants until the last larval stage. To determine the effect of the different treatments on survival, visible fungal infection, and melanization of *L. villosa* pupae, a Cox model was fitted to the data using the survival package ﻿(Version 2.2-16) in RStudio (Version 1.2.5042). Plots were obtained with the rms package ﻿(Version 5.1-4), making use of the Kaplan–Meier-estimator, and Adobe Illustrator (Adobe, Version 14.1, CC 2020).

### Chemical extraction and quantification of lagriamide

*L. villosa* specimens (Table S6) were collected in the field or recovered in the lab as offspring of field-collected females in the case of eggs and larvae of the two first instars. Eggs were extracted as a fraction of the clutch including up to 60 eggs. First and second instar larvae were combined in multiple pools of up to 22 individuals for extraction. Field-collected larvae, pupae, and exuviae were extracted individually. For female adult specimens, the ovipositor and both accessory glands were dissected. For adult males, the full reproductive system was dissected. Replicate numbers for each sample type are indicated in Table S6. Each sample was extracted in a known amount of methanol (200–600 μL) for at least 24 h. Extracts were kept at 10 °C until further processing. Crude extracts were analyzed by LC-MS on an Exactive Orbitrap High Performance Benchtop LC-MS (Thermo Fisher Scientific) with an electron spray ion source and an Accela HPLC System, C18 column (Betasil C18 5 µm, 150 × 2.1 mm, Thermo Fisher Scientific), solvents: acetonitrile and water (both supplemented with 0.1% formic acid), flow rate: 0.2 mL min^−1^; program: hold 1 min at 5 % acetonitrile, 1–16 min 5–98% acetonitrile, hold 3 min 98% acetonitrile. Lagriamide amounts were measured by integration of the peak areas of the extracted mass traces (*m/z*  =  747.4769 – 747.4843 [M-H]^−^) (ICIS integration algorithm in XCalibur, Thermo Fisher Scientific).

### Lagriamide localization by AP-SMALDI-HR MSI experiments

*L. villosa* specimens were collected in the field or recovered in the lab as offspring of field-collected females in the case of eggs and exuviae. Aposymbiotic individuals were collected from a culture of the 6^th^ lab generation. All samples were analyzed using an atmospheric pressure—scanning microprobe matrix-assisted laser desorption ionization (AP-SMALDI10, TransMIT, Gießen, Germany) ion source equipped with a UV (337 nm) nitrogen laser (LTB MNL-106, LTB, Germany). AP-SMALDI10 was coupled to a mass spectrometer Q‐Exactive Plus (Thermo Fisher Scientific, Bremen, Germany) providing high‐resolution mass spectra. Raw MS data were collected via Xcalibur software v.2.8 Build 2806 (Thermo Fisher Scientific), while the acquisition of spatial scans, predefined by the user in *x*‐ and *y*‐directions as a rectangular sample region, was controlled by the Master Control Program (TransMIT [82]).

Sample heterogeneity required minor changes in specific parameters of the AP-SMALDI-HR MSI method. Therefore, egg samples (cryosections and intact eggs) were imaged with a step size of 10 - 15 μm. Exuviae samples were analyzed based on their size using 25 μm - 45 μm. Sagittal cryosections of pupae and larvae sections were analyzed with a step size of 30 μm. The lagriamide standard (30% DMSO at a concentration of 168 ng/µL) was pipetted on hexane extracted fine paper tissue in a volume of 1 μL/mm^2^. All samples were imaged with the number of laser shots per spot set to 30 (approximately 1.1 μJ × shot^−1^ to analyze the egg samples; 1.0 μJ × shot^−1^ for exuviae samples and 1.2 μJ × shot^−1^ for the pupae and larvae sections) within a laser frequency of 60 Hz. A mass range was set from *m/z* 100 to *m/z* 1000 with a resolving power of 140,000. AP-SMALDI-HR MS profiling of lagriamide standard was performed in positive ion mode with several laser shots per spot set to 30 (approximately 13 μJ × shot^−1^) within a laser frequency of 60 Hz, a mass range was set from *m/z* 700 to *m/z* 850 with a resolving power of 280,000. Ion intensity maps of selected *m/z* values were generated using a Mirion 3D V3.3.64.13 software package [82] with an *m/z* width of 0.01 u. Ion maps were normalized separately to the highest intensity for each ion species. All acquisitions were performed in positive polarity and in laser desorption ionization mode (LDI) without applying the MALDI matrix to the sample.

Samples were prepared as follows: exuviae samples were cut open along the dorsal posterior region to flatten them on a double-sided adhesive tape (3 M, Maplewood, MN, USA) using a fine tweezer (Fig. S9a, b). This was attached to a microscope glass slide (25 mm × 15 mm, Thermo Scientific, Menzel‐Gläser, VWR, Darmstadt, Germany) and finally fixed on the AP-SMALDI metal target. Intact eggs were carefully picked up from a clutch using a clean tweezer and fixed on a double-sided tape attached to a microscope glass slide (25 mm × 15 mm). Glass slides with egg samples were mounted on an AP-SMALDI metal target and promptly placed into the AP-SMALDI ion chamber to be analyzed. Because of the conditions in the ion chamber and the nature of the egg samples, a negative effect of drying and shape distortion was observed (Fig. S9c, d). For sectioning, samples were embedded in O.C.T. medium (Tissue‐Tek O.C.T. Compound; Sakura Finetek, Torrance, CA, USA), frozen in liquid nitrogen, and cut into 30 µm sections using a Leica cryomicrotome (Leica CM1850, Leica Mikrosysteme Vertrieb GmbH, Germany). Fresh sections were transferred from a cryomicrotome chamber (–20 °C) into a desiccator (filled with potassium hydroxide as a drying agent) to dry without a vacuum for 20 min.

The identity of acquired lagriamide-related ions from *L. villosa* samples, which was based on exact mass, was supported by best matching chemical formulas and isotope patterns generated via Qual Browser/Xcalibur software 3.0.63. This enabled to putatively assign the peaks to lagriamide (C_41_H_68_N_2_O_10_) adducts (∆ error <1 ppm), which was confirmed with AP-SMALDI-HR MS profiling of a lagriamide standard (Fig. S9e). In total, 3 µL of the lagriamide standard (30% DMSO at concentration 168 ng/µL) was pipetted in three parts onto small pieces of tissue paper glued to a clean microscope glass slide. Except for the potassium adduct, which was predominantly found in MS/MSI datasets, ions representing the [M + H]^+^ or [M + Na]^+^ adducts were detected. Presence of all three lagriamide adducts was demonstrated on an extract obtained from an *L. villosa* larval exuvia (Fig. S9f).

### In vitro assays for lagriamide antimicrobial activity

Antimicrobial activity of lagriamide was studied by agar diffusion assays against *Bacillus thuringiensis* DSM2048, *Beauveria bassiana* ST000047, and *Metarhizium anisopliae* STH00420 (ATCC 24942). For the fungal assays, fifty microliters of a solution of lagriamide (168 ng/µL in 30% DMSO in methanol) were filled in agar holes of 9 mm diameter (malt extract agar, Merck, seeded with a spore suspension). After incubation at 23 °C for 4 d, the inhibition zone was evaluated. For the antibacterial assay, nutrient agar plates (Standard I nutrient agar, Merck) seeded with the bacterial culture were used and incubation took place at 30 °C. The inhibition zones were determined after 24 h.

## Supplementary information

Supplementary Information

## Data Availability

Raw sequence data generated in this study and used for 16 S rRNA gene bacterial community profiling of *L. villosa* life stages is available in the GenBank SRA database under the BioProject accession number PRJNA790999.
