# Supplementary material for: Bacterial ectosymbionts in cuticular organs chemically protect a beetle during molting stages
Source: ISME J. 2022 Sep 2;16(12):2691–701. doi: 10.1038/s41396-022-01311-x (PMC9666510; doi:10.1038/s41396-022-01311-x)
Supplement: Supplementary file 1 — Supplementary Information [file 41396_2022_1311_MOESM1_ESM.docx]

**Supplementary information**

**
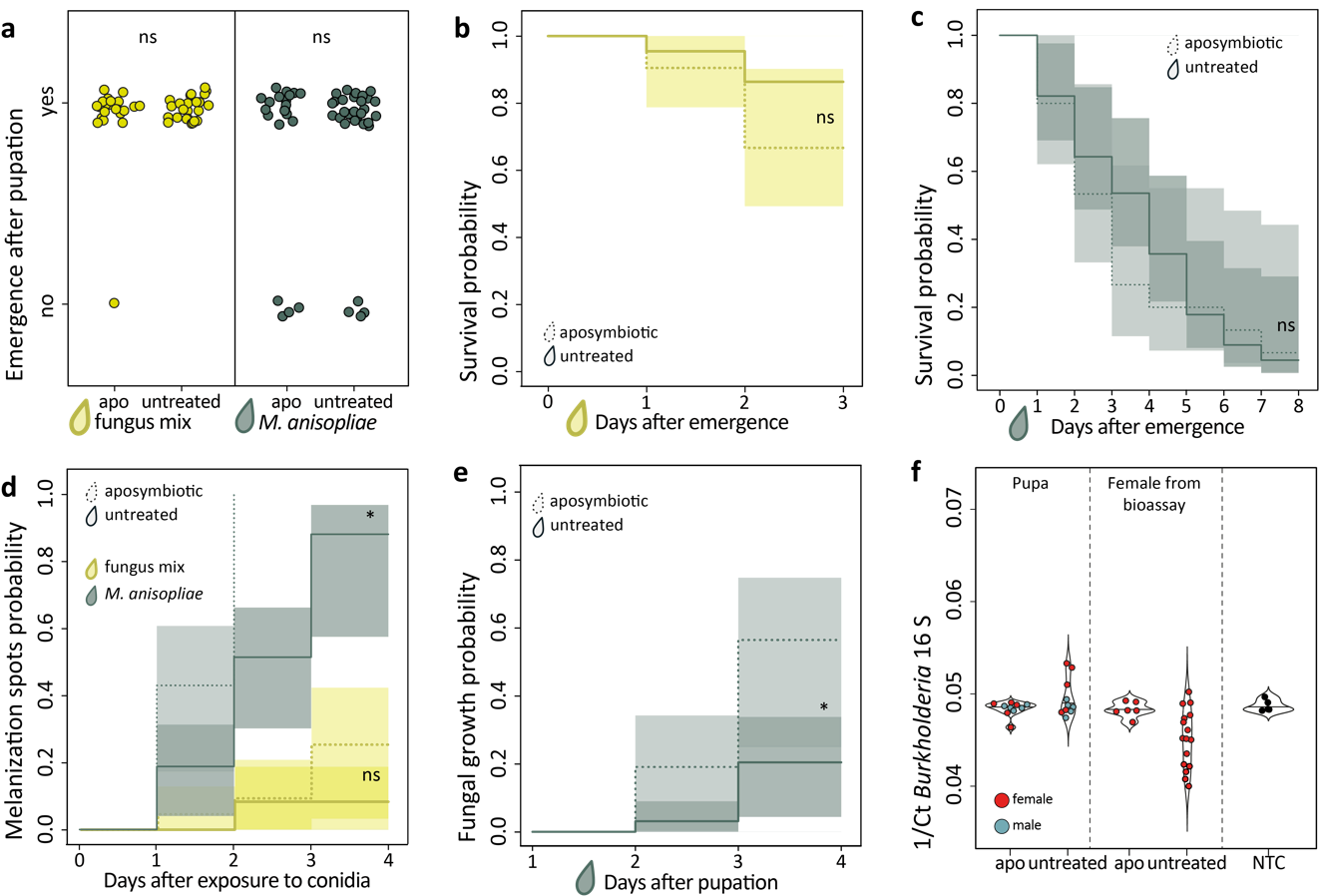
**

**Figure S1: Bacterial symbionts reduce growth of pathogenic fungi on *L. villosa* pupae.** Single pupae with (untreated, solid lines) and without symbionts (aposymbiotic, dotted lines) were exposed to different fungal pathogens (fungus mix in yellow or *M. anisopliae* in dark green) and were monitored single-blind. a) Emergence rate after pupation according to fungal treatment and symbiont infection status. b) Survival probability of aposymbiotic and untreated adults after infection by a fungus mix (10^6^ conidia of each *B. bassiana*, *M. anisopliae* and *P. lilacinum*) during pupation. c) Survival probability of aposymbiotic and untreated adults after an infection of 10^6^ conidia of *M. anisopliae* and additional topical application of 106 conidia on the dorsal thorax. d) Melanization probability of aposymbiotic and untreated pupae in response to different fungus treatments. Black lines indicate presence of a fungus mix, green lines indicate presence of *M. anisopliae*. e) Visible fungal growth on the pupae infected with *M. anisopliae* during pupation. For b - e statistically significant differences from Cox mixed effects model are indicated as: **p* < 0.05, ns= *p* > 0.05. Estimated survival curves (Kaplan–Meier) and the corresponding confidence intervals are shown. f) Symbiont presence of aposymbiotic and untreated pupae and adult females was assessed using qPCR. These individuals belong to the same batch as pupae used in the bioassays but were not exposed to fungus. Females correspond to individuals from the bioassay. The results show *Burkholderia* presence in 3 out of 10 untreated pupae, and symbiont absence in all females after the bioassay.


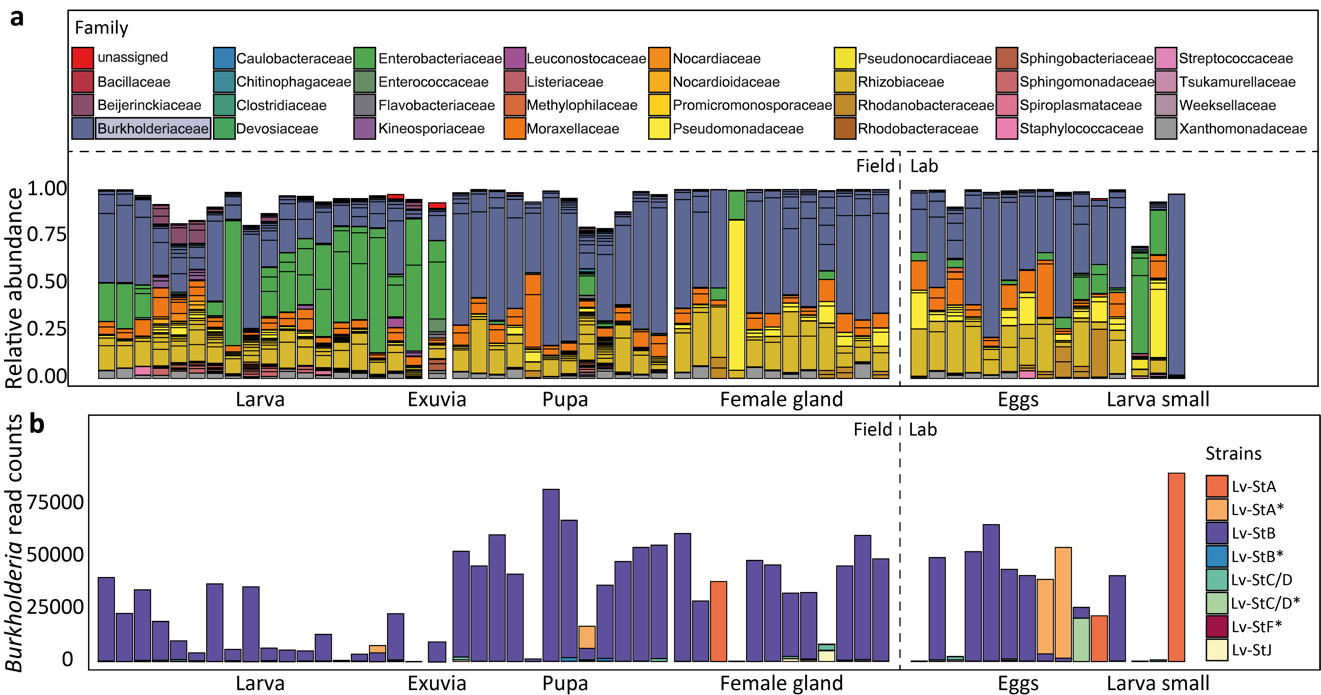


**Figure S2: Relative abundance of bacterial families and read counts of *Burkholderia* strains across *L. villosa* life stages.** a) *Lagria villosa* bacterial community composition of different life stages shown at bacterial family level. Burkholderiaceae was abundant in most individuals of all life stages. In larvae, Enterobacteriaceae and Rhizobiaceae were also predominant and are most likely associated to the gut. b) Read counts per sample of *Burkholderia* strains across different life stages. (*) in legend denotes pairwise identity above 98% but below 100%.


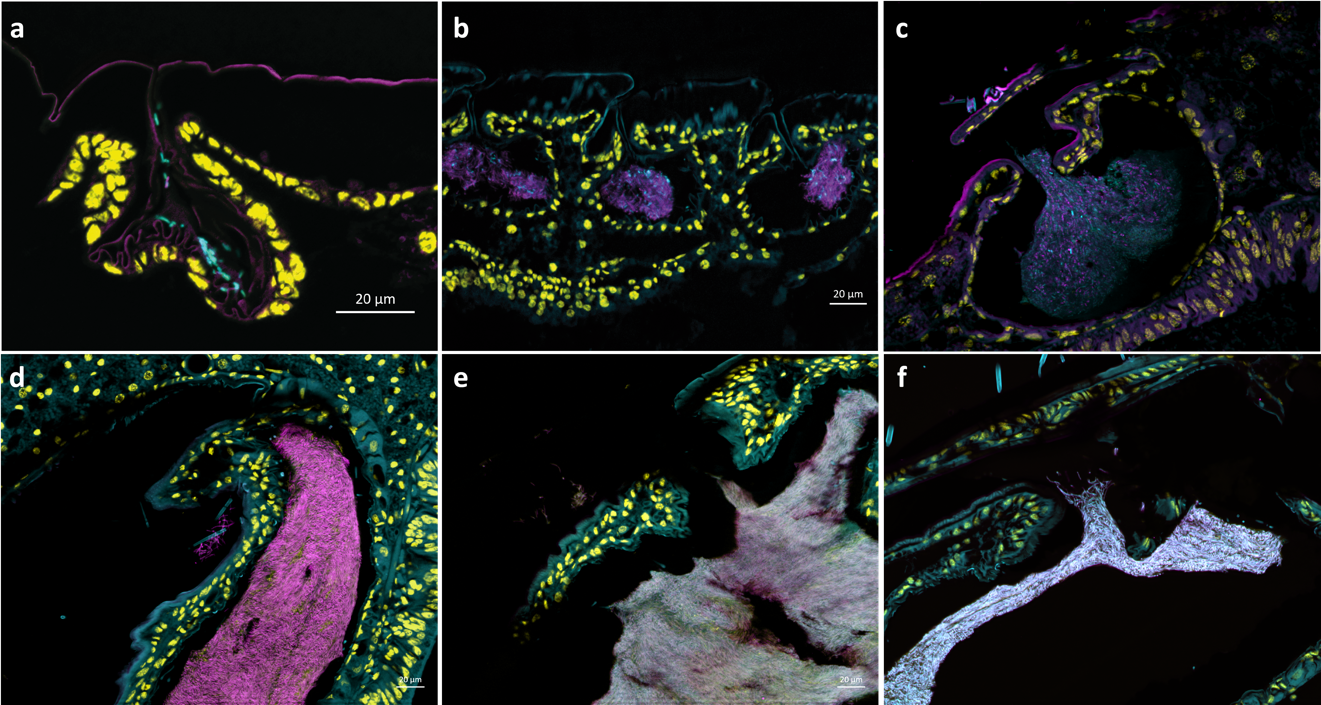


**Figure S3: Presence of other bacterial symbionts or other *Burkholderia* strains in the symbiotic organs of *L. villosa* larvae.** a) Sagittal section of an early L1 larva showing very few *Burkholderia* cells (magenta) among other bacteria (cyan). b) Sagittal section of an L1 larva showing few other *Burkholderia* strains (cyan) in between Lv-StB (magenta). c) Sagittal section of a medium-sized larva showing *Burkholderia* cells (magenta) along with other bacteria (cyan) being released through the opening to the outer surface. d-f) Sagittal sections through organs of older larvae showing higher abundance of *Burkholderia* (magenta) among all bacteria (cyan), while most of the cells are labeled with both probes (violet-white).


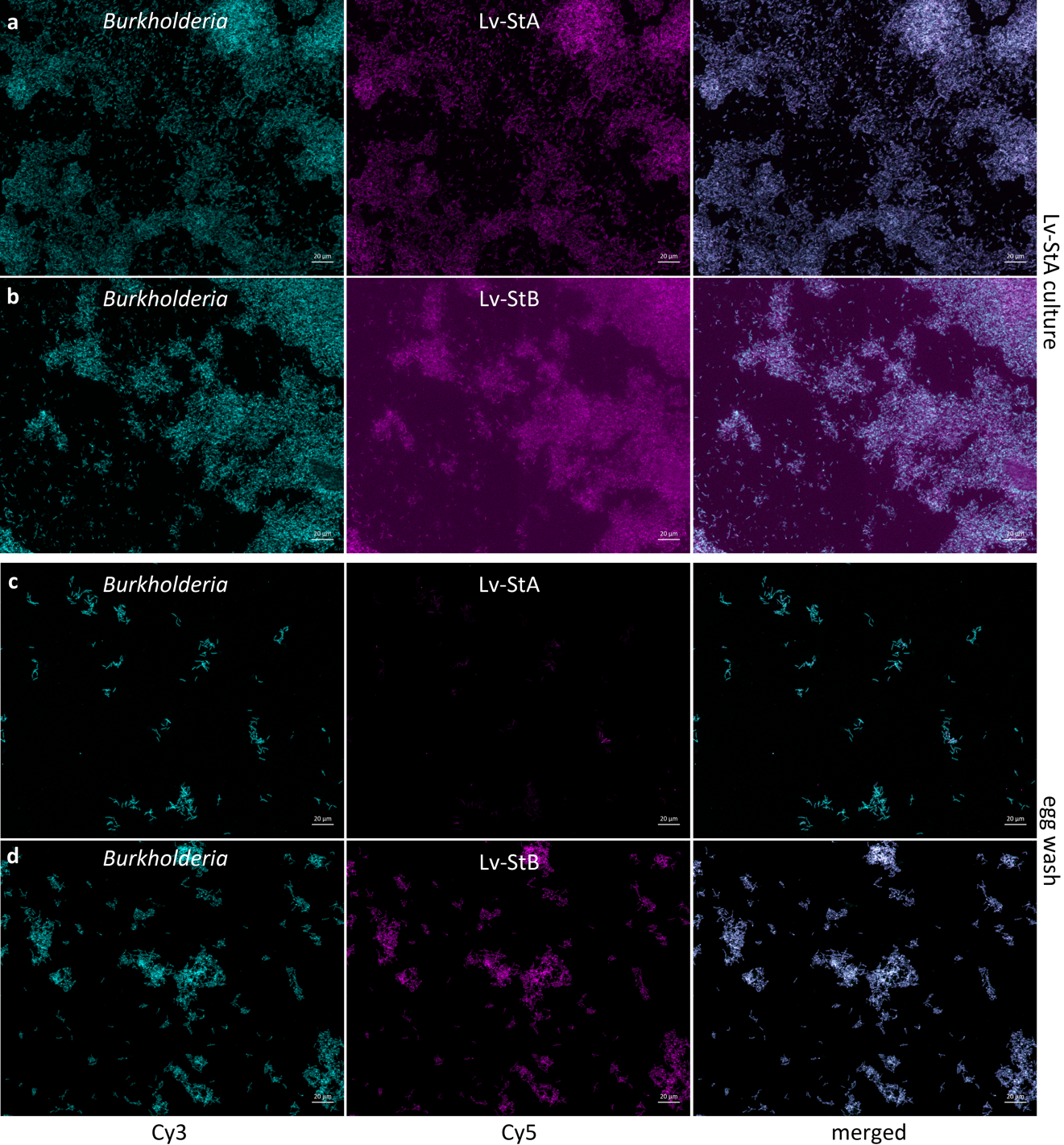


**Figure S4: Specificity of FISH probes used for Lv-StB and Lv-StA staining.** *Burkholderia*-specific staining in Cy3 (left panels) is depicted in cyan while Lv-StB and Lv-StA-specific staining in Cy5 (middle panels) is shown in magenta and a merged staining is shown in violet-white (right panel). a) Culture of Lv-StA showing a clear signal for the general *Burkholderia* and Lv-StA-specific probe in all the cells. b) Culture of Lv-StA showing clear signal for the *Burkholderia*-specific probe, and low signal to noise ratio with the Lv-StB probe. c) Egg wash of *L. villosa* eggs showing labeling for single cells with the *Burkholderia* probe and only a few single cells with the Lv-StA probe. d) Egg wash of *L. villosa* eggs stained with the Lv-StB-specific probe showing labeling of all cells. a, b) Images were taken with the same exposure times. The white labels on the upper region of each panel refer to the FISH-probe used, while the y-axis labeling shows the sample and the x-axis label the fluorescence channel.


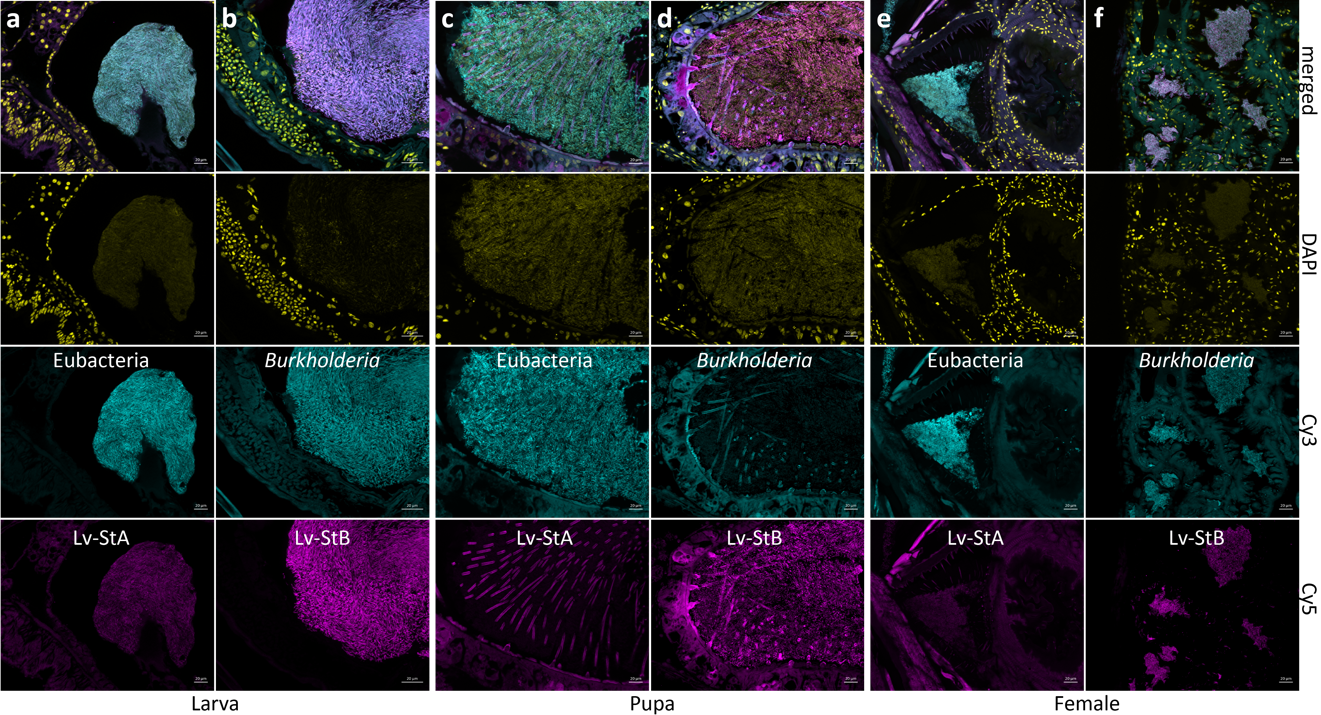


**Figure S5: Lv-StA and Lv-StB presence on *L. villosa* sections.** Sagittal sections of field individuals (same as in Figure 4) were used to compare the FISH signal of the strains-specific probes. The first row shows the merged image of the channels. The following rows show the individual channels corresponding to DAPI (yellow), probes for Eubacteria (a,c,e) or *Burkholderia* (b,d,f) labeled with Cy3 (cyan) and specific probes designed for Lv-StA (a,c,e) or Lv-StB (b,d,f) labeled with Cy5 (magenta). a,b) Larval organ (Figure 4 e) showing intense signal for Eubacteria, *Burkholderia* and Lv-StB, in contrast to the Lv-StA probe. c,d) Pupal organ (Figure 4 f) showing clear signal for the Eubacteria and Lv-StB probes, moderate signal for *Burkholderia* and no signal for Lv-StA. e,f) Female reproductive system showing strong labeling for Eubacteria, *Burkholderia* and Lv-StB, as opposed to the Lv-StA probe. In all *L. villosa* sections, there was usually minor autofluorescence for the host tissue in the Cy3 channel, except for strong cuticular structures, which is usual for this wavelength range. The Cy5 channel usually showed no autofluorescence of the host tissue when specific labeling is visible for the symbionts. The white labels on the upper region of each panel refer to the FISH-probe used, while the y-axis labeling shows the fluorescence channel and the x-axis label shows the sample.


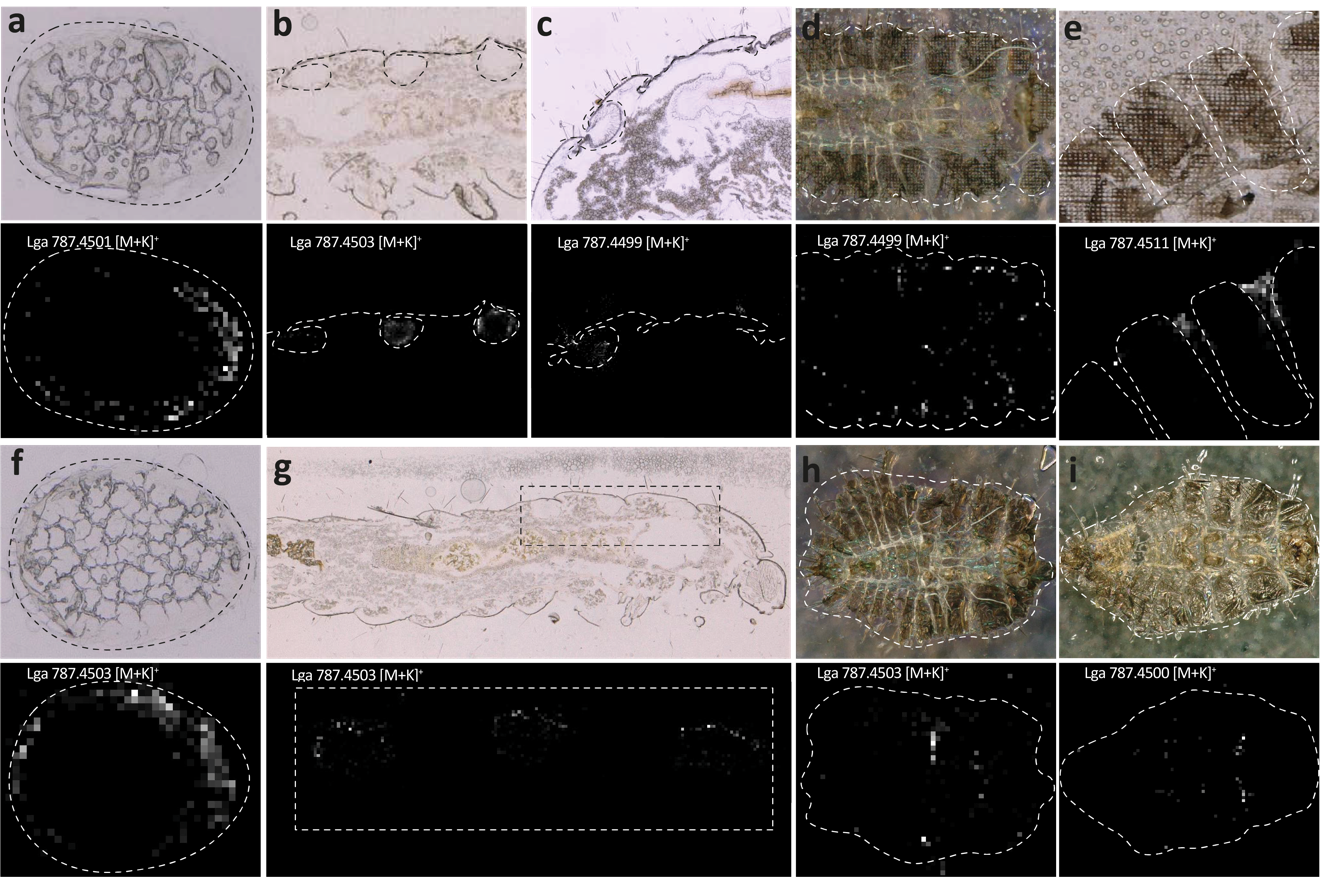


**Figure S6: Lagriamide detection on *L. villosa* tissue using AP-SMALDI-HR MSI**. Micrographs and the corresponding MSI images from Figure 5 c-h of sections through a) an egg, b) a larva, c) a pupa, and on d) a whole exuvia, and e) parts of an exuvia. Additional replicates of f) an egg section, g) a larval section and h and i) whole larval exuviae.


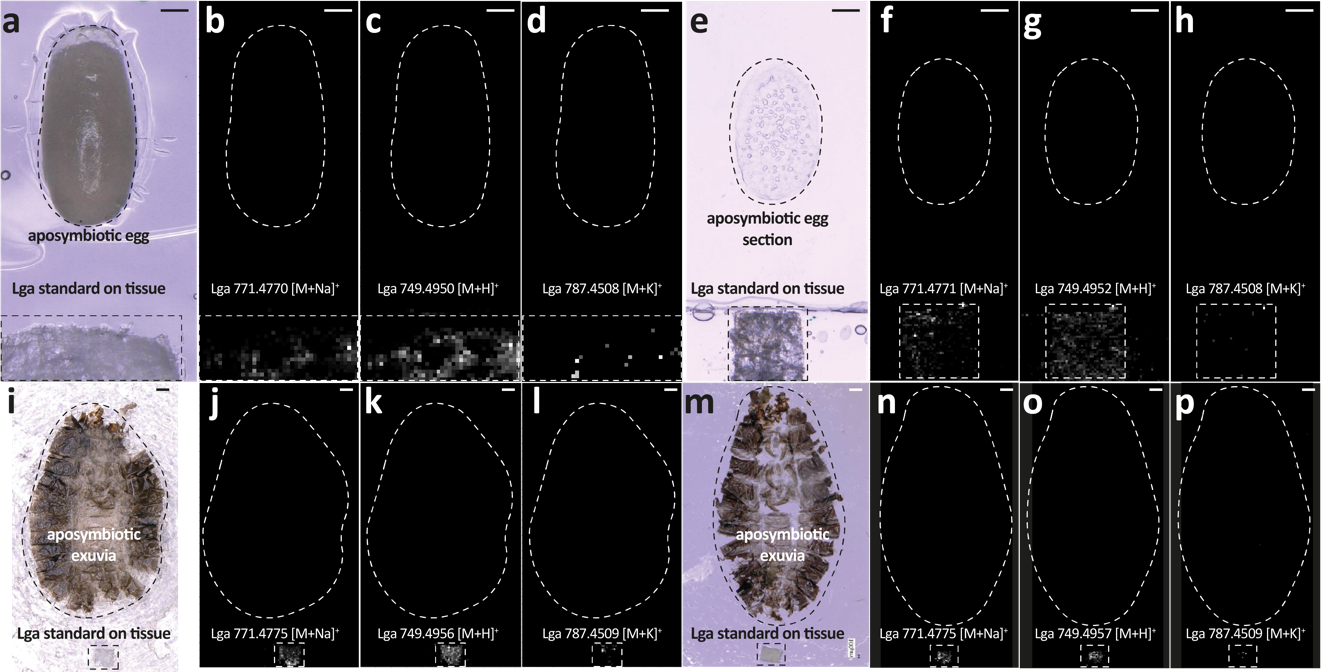


**Figure S7: Positive (lagriamide standard) and negative controls (aposymbiotic *L. villosa* samples) for lagriamide detection using AP-SMALDI-HR MSI.** Micrographs and the corresponding MSI images showing [M+Na]^+^, [M+H]^+^ and [M+K]^+^ adducts of lagriamide on negative controls of *L. villosa* samples without Lv-StB (outlined ovals) and positive controls of pure lagriamide on tissue (outlined rectangles). a-d) Intact aposymbiotic egg. e-h) Aposymbiotic egg section. i-p) Two flattened aposymbiotic exuviae from larvae. Scale bars 100 µm for the top row and 500 µm for the bottom row.


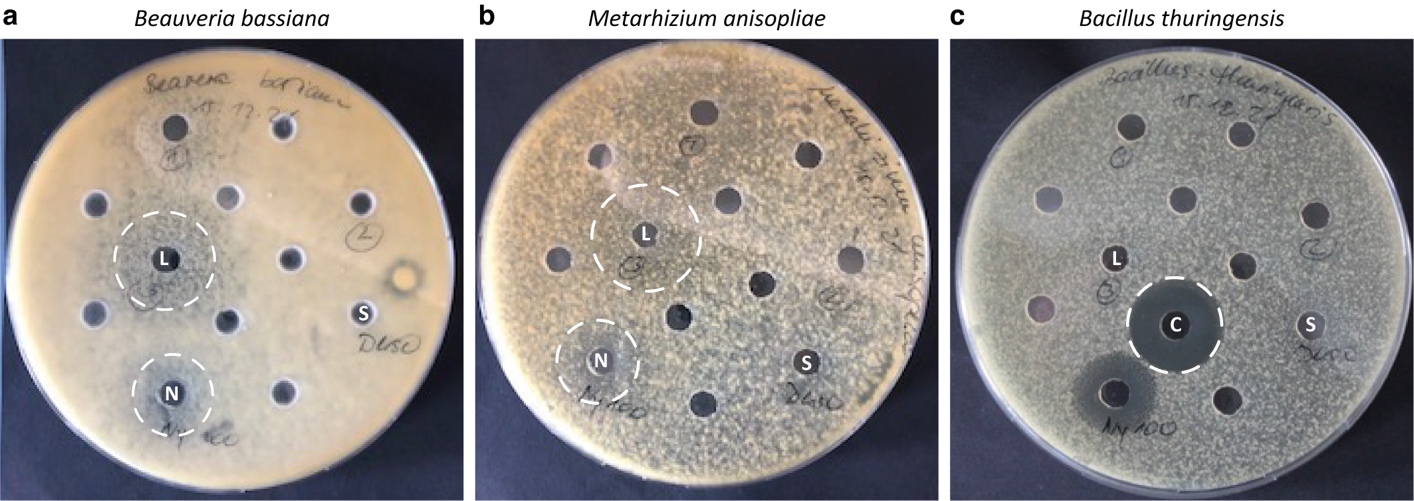


**Figure S8: *In vitro* activity profiling of lagriamide.** a) Inhibition zone of 24 mm by lagriamide (L) against *B. bassiana*. b) Inhibition zone of 18 mm by lagriamide (L) against *M. anisopliae*. c) No inhibition by lagriamide (L) against *B. thuringensis*. L=lagriamide, N=nystatin, C= ciprofloxacin, S=solvent (DMSO).


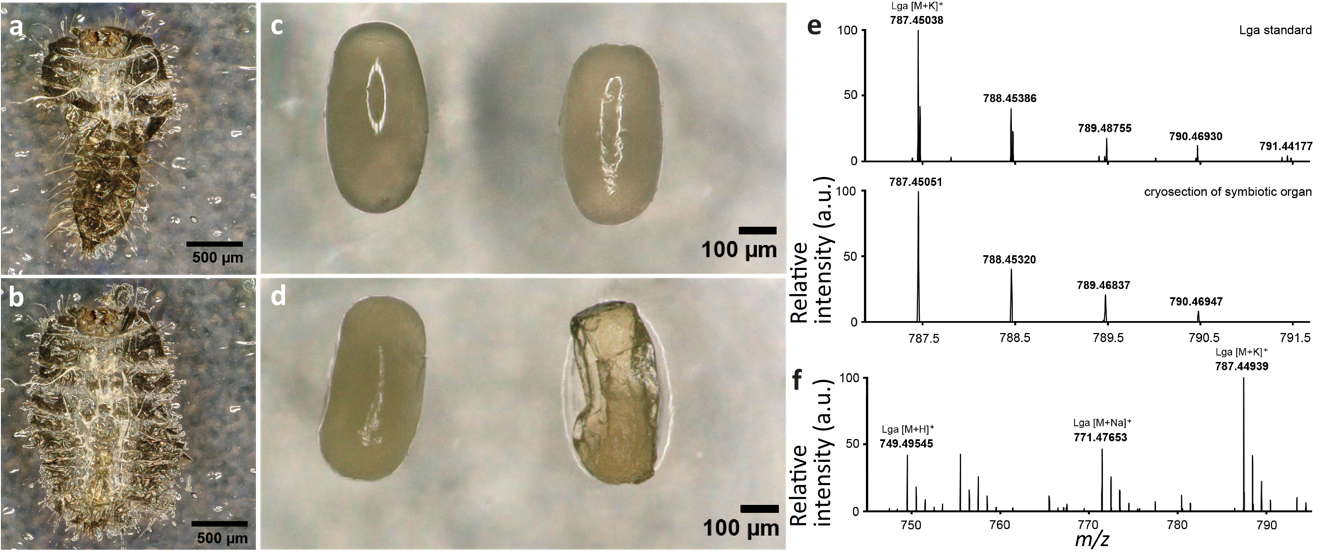


**Figure S9: AP-SMALDI-HR MSI experimental procedure.** a-d) Fixing process of *L. villosa* samples on double sided tape: a) Exuvia before flattening and b) after flattening. c) Intact eggs before and d) after 30 min inside AP-SMALDI ion chamber (+30 °C), egg at the right side was analyzed via AP-SMALDI-HR MSI. e) AP-SMALDI-HR MS profiling of lagriamide standard (upper MS spectrum) in comparison with a cryosection of a symbiotic organ of a *L. villosa* larva (lower MS spectrum). Lagriamide was detected mainly as a potassium adduct at *m/z* 787.450 [M+K]+. f) AP-SMALDI-HR MS profiling of *L. villosa* larva exuviae crude methanolic extract. Typical lagriamide adducts formed during AP-SMALDI-HR imaging and profiling experiments.

**Table S1: Experimental set-up and replicate numbers of bioassays including differently treated *L. villosa* life stages and different fungi**

| Life stage | Experiment | Fungus | Number of applied conidia | Number of clutches | Treatment | Number of individuals |
| --- | --- | --- | --- | --- | --- | --- |
| Larvae | Survival | *P. lilacinum* | 7.5 x 10^3^ | 8 | aposymbiotic | 111 |
|  |  |  |  |  | untreated | 111 |
|  |  |  |  |  | reinfected-egg wash | 103 |
|  |  |  |  |  | reinfected-LvStA | 127 |
|  | Survival & Fungal growth | *B. bassiana* | 10^6^ | 4 | aposymbiotic | 70 |
|  |  |  |  |  | reinfected-egg wash | 69 |
|  |  | *M. anisopliae* | 10^6^ | 4 | aposymbiotic | 68 |
|  |  |  |  |  | reinfected-egg wash | 66 |
|  |  | No-fungus-control | - | 4 | aposymbiotic | 65 |
|  |  |  |  |  | reinfected-egg wash | 66 |
| Pupae | Melanization & fungal growth | fungus mix | 10^6^ | 6 | aposymbiotic | 22 |
|  |  |  |  |  | untreated | 24 |
|  |  | *M. anisopliae* | 10^6^ & topically applied 10^6^ | 6 | aposymbiotic | 21 |
|  |  |  |  |  | untreated | 32 |
| Adults | Survival | *M. anisopliae* | 10^6^ | 6 | aposymbiotic | 17 |
|  |  |  |  |  | untreated | 28 |

**Table S2: Statistical analysis of the larval bioassay against *P. lilacinum* using a Cox mixed-effects model fit by maximum likelihood with survival as output, treatment as fixed effect and random intercepts per clutch and per year.**

| Cox mixed-effects model fit by maximum likelihood |  | NULL | Integrated | Fitted |  |  |
| --- | --- | --- | --- | --- | --- | --- |
|  | Log-likelihood | -789.3066 | -775.1302 | -768.8992 |  |  |
|  |  | Chisq | df | p | AIC | BIC |
|  | Integrated loglik | 28.35 | 5.00 | 3.1051e-05 | 18.35 | 3.86 |
|  | Penalized loglik | 40.81 | 7.64 | 1.6216e-06 | 25.53 | 3.39 |
| Random effects | | Std Dev | Variance |  |  |  |
|  | Clutch | 0.3590747895 | 0.1289347044 |  |  |  |
|  | Year | 0.0199984204 | 0.0003999368 |  |  |  |
| Fixed coefficients | | coef | coef(exp) | se(coef) | z | p |
| aposymbiotic | untreated | 0.7642137 | 0.4656999 | 0.2436558 | 3.14 | 1.7e-03 |
| aposymbiotic | reinfected-egg wash | 1.1607199 | 0.3132606 | 0.2791207 | 4.16 | 3.2e-05 |
| aposymbiotic | reinfected-StA | 0.4840728 | 0.6162683 | 0.2129933 | 2.27 | 2.3e-02 |
| untreated | reinfected-egg wash | 0.3965062 | 0.6726661 | 0.3111214 | 1.27 | 0.2000 |
| untreated | reinfected-StA | 0.2801409 | 1.3233163 | 0.2520390 | 1.11 | 0.2700 |
| reinfected-egg wash | reinfected-StA | 0.6766471 | 1.967271 | 0.2852862 | 2.37 | 1.8e-02 |

**Table S3: Statistical analysis of the larval bioassay including no-fungus-control, *B. bassiana* and *M. anisopliae* using Cox mixed-effects models fit by maximum likelihood with survival as output, treatment and fungus as fixed effect and a random intercept per clutch.** In addition, Cox mixed-effects models within the single fungal treatments were carried out with treatment as fixed effect and a random intercept per clutch.

| Cox mixed-effects model fit by maximum likelihood |  | NULL | Integrated | Fitted |  |  |
| --- | --- | --- | --- | --- | --- | --- |
|  | Log-likelihood | -1076.26 | -966.8294 | -962.2197 |  |  |
|  |  | Chisq | df | p | AIC | BIC |
|  | Integrated loglik | 218.86 | 4.00 | 0 | 210.86 | 197.87 |
|  | Penalized loglik | 228.08 | 5.66 | 0 | 216.76 | 198.38 |
| Random effects | | Std Dev | Variance |  |  |  |
|  | Clutch | 0.4110391 | 0.1689531 |  |  |  |
| Fixed coefficients | | coef | coef(exp) | se(coef) | z | p |
| aposymbiotic | reinfected-egg wash | -0.4653203 | 0.6279339 | 0.1467154 | -3.17 | 1.5e-03 |
| *B. bassiana* | *M. anisopliae* | 1.0749012 | 2.9297034 | 0.1576536 | 6.82 | 9.2e-12 |
| *B. bassiana* | no-fungus-control | -2.2907314 | 0.1011924 | 0.3411659 | -6.71 | 1.9e-11 |
| *M. anisopliae* | no-fungus-control | 3.3656326 | 28.9518052 | 0.3347164 | 10.06 | 0.0e+00 |
| Cox mixed-effects model fit by maximum likelihood  No-fungus-control |  | NULL | Integrated | Fitted |  |  |
|  | Log-likelihood | -48.11166 | 48.10818 | -48.0339 |  |  |
|  |  | Chisq | df | p | AIC | BIC |
|  | Integrated loglik | 0.01 | 2.00 | 0.99652 | -3.99 | -4.60 |
|  | Penalized loglik | 0.16 | 1.07 | 0.72143 | -1.99 | -2.31 |
| Random effects | | Std Dev | Variance |  |  |  |
|  | Clutch | 0.10004711 | 0.01000942 |  |  |  |
| Fixed coefficients | | coef | coef(exp) | se(coef) | z | p |
| aposymbiotic | reinfected-egg wash | 0.03887024 | 1.039636 | 0.6326311 | 0.06 | 0.95 |
| Cox mixed-effects model fit by maximum likelihood  *B. bassiana* |  | NULL | Integrated | Fitted |  |  |
|  | Log-likelihood | -302.1836 | -290.7721 | -286.0536 |  |  |
|  |  | Chisq | df | p | AIC | BIC |
|  | Integrated loglik | 22.82 | 2.00 | 1.1068e-05 | 18.82 | 14.44 |
|  | Penalized loglik | 32.26 | 3.68 | 1.1417e-06 | 24.90 | 16.84 |
| Random effects | | Std Dev | Variance |  |  |  |
|  | Clutch | 0.7435752 | 0.5529041 |  |  |  |
| Fixed coefficients | | coef | coef(exp) | se(coef) | z | p |
| aposymbiotic | reinfected-egg wash | -0.7759086 | 0.4602854 | 0.2600352 | -2.98 | 0.0028 |
| Cox mixed-effects model fit by maximum likelihood  *M. anisopliae* |  | NULL | Integrated | Fitted |  |  |
|  | Log-likelihood | -474.072 | -470.218 | -467.7515 |  |  |
|  |  | Chisq | df | p | AIC | BIC |
|  | Integrated loglik | 7.71 | 2.0 | 0.0211960 | 3.71 | -1.76 |
|  | Penalized loglik | 12.64 | 2.9 | 0.0049451 | 6.85 | -1.07 |
| Random effects | | Std Dev | Variance |  |  |  |
|  | Clutch | 0.25010300 | 0.06255151 |  |  |  |
| Fixed coefficients | | coef | coef(exp) | se(coef) | z | p |
| aposymbiotic | reinfected-egg wash | -0.4217744 | 0.655882 | 0.1892313 | -2.23 | 0.026 |

**Table S4: Statistical analysis of the larval bioassay including no-fungus-control, *B. bassiana* and *M. anisopliae* using Cox mixed-effects models fit by maximum likelihood with fungal infestation as output, treatment and fungus as fixed effect and a random intercept per clutch.** In addition, Cox mixed-effects models within the single fungal treatments were carried out with treatment as fixed effect and a random intercept per clutch. Having only ceros in individual groups impedes running this kind of model. Therefore, given that in the no-fungus-control treatment none of the aposymbiotic and natural-reinfected individuals originally showed signs of fungal growth, a single fungus-positive sample was artificially added to each of these two groups.

| Cox mixed-effects model fit by maximum likelihood |  | NULL | Integrated | Fitted |  |  |
| --- | --- | --- | --- | --- | --- | --- |
|  | Log-likelihood | -822.0822 | -675.8562 | -669.1933 |  |  |
|  |  | Chisq | df | p | AIC | BIC |
|  | Integrated loglik | 292.45 | 4.00 | 0 | 284.45 | 272.52 |
|  | Penalized loglik | 305.78 | 5.9 | 0 | 293.98 | 276.37 |
| Random effects | | Std Dev | Variance |  |  |  |
|  | Clutch | 0.9624150 | 0.9262427 |  |  |  |
| Fixed coefficients | | coef | coef(exp) | se(coef) | z | p |
| aposymbiotic | reinfected-egg wash | -1.393525 | 0.248198776 | 0.1868122 | -7.46 | 8.7e-14 |
| *B. bassiana* | *M. anisopliae* | -1.263624 | 0.282627988 | 0.1823727 | -6.93 | 4.2e-12 |
| *B. bassiana* | no-fungus-control | 4.336962 | 76.4748646 | 0.7272239 | 5.96 | 2.5e-09 |
| *M. anisopliae* | no-fungus-control | -5.600586 | 270.5848957 | 0.7298289 | -7.67 | 1.7e-14 |
| Cox mixed-effects model fit by maximum likelihood  No-fungus-control |  | NULL | Integrated | Fitted |  |  |
|  | Log-likelihood | 0 | 0 | 0 |  |  |
|  |  | Chisq | df | p | AIC | BIC |
|  | Integrated loglik | 0 | 2 | 1 | -4 | Inf |
|  | Penalized loglik | 0 | 1 | 1 | -2 | Inf |
| Random effects | | Std Dev | Variance |  |  |  |
|  | Clutch | 2e-02 | 4e-04 |  |  |  |
| Fixed coefficients | | coef | coef(exp) | se(coef) | z | p |
| aposymbiotic | reinfected-egg wash | 0 | 1 | 0 | NaN | NaN |
| Cox mixed-effects model fit by maximum likelihood  *B. bassiana* |  | NULL | Integrated | Fitted |  |  |
|  | Log-likelihood | -240.7052 | -209.7258 | -203.8468 |  |  |
|  |  | Chisq | df | p | AIC | BIC |
|  | Integrated loglik | 61.96 | 2.00 | 3.5194e-14 | 57.96 | 54.02 |
|  | Penalized loglik | 73.72 | 3.84 | 2.8866e-15 | 66.04 | 58.48 |
| Random effects | | Std Dev | Variance |  |  |  |
|  | Clutch | 1.304286 | 1.701162 |  |  |  |
| Fixed coefficients | | coef | coef(exp) | se(coef) | z | p |
| aposymbiotic | reinfected-egg wash | -1.812083 | 0.1633136 | 0.3258503 | -5.56 | 2.7e-08 |
| Cox mixed-effects model fit by maximum likelihood  *M. anisopliae* |  | NULL | Integrated | Fitted |  |  |
|  | Log-likelihood | -370.1209 | -349.1938 | -343.726 |  |  |
|  |  | Chisq | df | p | AIC | BIC |
|  | Integrated loglik | 41.85 | 2.00 | 8.1554e-10 | 37.85 | 32.83 |
|  | Penalized loglik | 52.79 | 3.79 | 6.8880e-11 | 45.22 | 35.72 |
| Random effects | | Std Dev | Variance |  |  |  |
|  | Clutch | 0.8185416 | 0.6700103 |  |  |  |
| Fixed coefficients | | coef | coef(exp) | se(coef) | z | p |
| aposymbiotic | reinfected-egg wash | -1.234547 | 0.2909667 | 0.2361363 | -5.23 | 1.7e-07 |

**Table S5: Characteristics and replicate numbers for specimens used to quantify *B. gladioli* Lv-StB across *L. villosa* life stages.**

| **Sample type** | **Replicates** | **Source** | **Content per replicate** |
| --- | --- | --- | --- |
| Larva | 12 | Field | Single individual |
| Larva late | 12 | Field | Single individual |
| Pupa | 6 | Field | Single individual |
| Female ovipositor + paired accessory glands | 5 | Field | Single individual, dissected organs |
| Fraction of early egg clutch | 32 | 1^st^ lab generation | Pools of 10-15 individuals from one clutch |
| Fraction of late egg clutch | 28 | 1^st^ lab generation | Pools of 6-15 individuals from one clutch |
| Larva L1 | 24 | 1^st^ lab generation | Pools of 9-15 individuals from one clutch |
| Larva early L2 | 25 | 1^st^ lab generation | Pools of 2-10 individuals from one clutch |
| Larva mid L2 | 24 | 1^st^ lab generation | Pools of 7-10 individuals from one clutch |
| Larva late L7 | 4 | 1^st^ lab generation | Single individual |
| Pupa early | 7 | 1^st^ lab generation | Single individual |
| Pupa late | 4 | 1^st^ lab generation | Single individual |

**Table S6: *L. villosa* specimens used for quantification of lagriamide throughout host development including replicate numbers and source environment.**

| **Sample type** | **Replicates** | **Source** | **Content per replicate** |
| --- | --- | --- | --- |
| Fraction of egg clutch | 27 | 1^st^ lab generation | 13–60 eggs, titer normalized per individual egg |
| Larva L1 | 8 | 1^st^ lab generation | 1^st^ instar, 20–22 individuals, titer normalized per individual |
| Larva L2 | 6 | 1^st^ lab generation | 2^nd^ instar, 6–20 individuals, titer normalized per individual |
| Larva (unknown instar) | 35 | Field | Single individual, unknown instar |
| Pupa | 18 | Field | Single individual |
| Pupa-adult exuvia | 14 | Field | Single individual |
| Female ovipositor + paired accessory glands | 6 | Field | Single individual, dissected organs |
| Male reproductive system | 3 | Field | Single individual, dissected organs |

**Table S7: Original localities of collected beetles and their use for this study.**

| **Year** | **State** | **Locality** | **Latitude** | **Longitude** | **Use** |
| --- | --- | --- | --- | --- | --- |
| 2020-2 | São Paulo | Perdobas, Cordeirópolis | 22°29'27.1"S | 47°26'04.7"W | Bioassay pupae |
| 2020 | São Paulo | Limeira | 22°38'20.4"S | 47°19'38.0"W | Bioassay larvae,  MALDI |
| 2020 | São Paulo | Cordeirópolis | 22°29'22.6"S | 47°23'45.6"W |  |
| 2020 | São Paulo | Santa Gertrudes | 22°27'56.6"S | 47°31'56.8"W |  |
| 2020 | São Paulo | Cordeirópolis | 22°29'14.6"S | 47°27'52.0"W |  |
| 2020 | São Paulo | Jaú | 22°15'53.6"S | 48°31'08.2"W |  |
| 2019-2 | São Paulo | Jundiaí | S23° 8' 3.732" | W46° 58' 47.352" | Bioassay larvae,  FISH,  MALDI |
| 2019-2 | São Paulo | Jundiaí | S23° 7' 42.06" | W46° 59' 25.296" |  |
| 2019-2 | São Paulo | Cordeirópolis | S22° 30' 11.376" | W47° 25' 40.08" |  |
| 2019-2 | São Paulo | Cordeirópolis | S22° 30' 13.32" | W47° 25' 28.092" |  |
| 2019-2 | São Paulo | Itirapina | S22° 15' 15.84" | W47° 50' 43.728" |  |
| 2019-2 | São Paulo | Brotas | S22° 16' 18.516" | W47° 56' 4.452" |  |
| 2019-2 | São Paulo | Brotas | S22° 17' 25.98" | W48° 3' 12.276" |  |
| 2019 | São Paulo | Cordeirópolis | S22° 29' 26.88" | W47° 25' 58.476" | Quantification of Lv-StB,  FISH,  MALDI |
| 2019 | São Paulo | Brotas | S22° 17' 25.98" | W48° 3' 12.276" |  |
| 2019 | São Paulo | Jaú | S22° 15' 49.896" | W48° 31' 12.396" |  |
| 2019 | São Paulo | Cordeirópolis | S22° 29' 40.776" | W47° 23' 48.192" |  |
| 2019 | São Paulo | Santa Gertrudes | S22° 27' 56.196" | W47° 31' 55.488" |  |
| 2019 | São Paulo | Cordeirópolis | S22° 30' 11.376" | W47° 25' 40.08" |  |
| 2018 | São Paulo | Jundiaí | -23°08'04.7040" | -046°58'53.5800" | Bacterial community profiling,  FISH,  MALDI |
| 2018 | São Paulo | Cordeirópolis | -22°30'13.3200" | -047°25'28.0920" |  |
| 2018 | São Paulo | Itirapina | -22°15'18.9000" | -047°51'00.1440" |  |
| 2018 | São Paulo | Guarapuã | -22°14'49.5960" | -048°17'47.9400" |  |
| 2018 | São Paulo | Jaú | -22°15'20.7720" | -048°33'27.2520" |  |
| 2018 | São Paulo | Santa Cruz da Conceição | -22°05'08.3760" | -047°25'17.2920" |  |
| 2018 | São Paulo | Santa Cruz da Conceição | -22°05'50.9280" | -047°25'16.1400" |  |
| 2018 | São Paulo | Pirassununga | -22°03'12.7440" | -047°32'05.3520" |  |
| 2018 | São Paulo | Brotas | -22°16'18.5160" | -047°56'04.4520" |  |
| 2017 | São Paulo | Itajú | -21°56'30.1920" | -048°51'23.5080" | Bacterial community profiling |
| 2017 | São Paulo | Jaú | -22°15'49.7880" | -048°31'10.2360" |  |
| 2017 | São Paulo | Jaú | -22°15'14.4000" | -048°33'49.4640" |  |
| 2017 | São Paulo | Jaú | -22°12'32.4720" | -048°36'35.4960" |  |
| 2017 | São Paulo | Itirapina | -22°16'03.3600" | -047°55'48.2880" |  |
| 2017 | São Paulo | Pirassununga | -22°03'12.7440" | -047°32'05.3520" |  |
| 2017 | Paraná | Ponta Grossa | -25°05'46.9644" | -050°02'55.6008" |  |
| 2017 | Paraná | Ponta Grossa | -25°05'35.2824" | -050°02'56.8392" |  |
